# TDP‐43 Aggregation: The Healthy‐Toxic Balance of the Prion‐Like Domain

**DOI:** 10.1002/advs.76119

**Published:** 2026-06-15

**Authors:** Luca Zangrando, Emanuele Buratti, Francesca Paron

**Affiliations:** ^1^ Life Science Department University of Trieste Trieste Italy; ^2^ Molecular Pathology International Centre for Genetic Engineering and Biotechnology Trieste Italy

**Keywords:** LLPS, neurodegeneration, prion‐like domain, protein aggregation, TDP‐43, therapeutic strategies

## Abstract

TAR DNA‐binding protein 43 (TDP‐43) is a ubiquitously expressed RNA‐binding protein that plays essential roles in RNA metabolism, including transcription, splicing, transport, and stability. Pathological TDP‐43 aggregates have become a defining hallmark of neurodegenerative diseases such as amyotrophic lateral sclerosis (ALS) and a large subset of frontotemporal lobar degeneration (FTLD). In the last decade, increasing evidence has challenged the initial thought of TDP‐43 condensates as a purely pathological event, highlighting instead the physiological relevance of reversible self‐association, polymerization and liquid‐liquid phase separation (LLPS) in regulating TDP‐43 functions. In this review, we provide an integrated overview of the structural determinants governing TDP‐43 two‐faced polymerization, with a particular focus on the prion‐like domain and its parallelism with prion proteins. Indeed, while physiological assemblies support normal RNA processing, the dysregulation of LLPS by either disease‐associated mutations, altered RNA‐binding, aberrant post‐translational modifications, or proteolytic cleavage can promote the transition toward irreversible, pathogenic aggregates. Finally, we summarize strategies aimed at eliminating TDP‐43 aggregates or modulating its phase‐separation behavior. Altogether, this review frames TDP‐43 polymerization in both healthy and pathological conditions, offering a prion‐like centered view of TDP‐43 proteinopathies.

## Graphical abstract

TDP‐43 function relies on a delicate balance between reversible phase‐separated states and irreversible aggregation. Under physiological conditions, TDP‐43 forms dynamic droplets and oligomers that support normal cellular functions. In pathological contexts, this balance shifts toward aberrant aggregation, leading to toxic species. Therapeutic strategies aim to restore or maintain this equilibrium, preventing toxic aggregation while preserving functional assemblies.

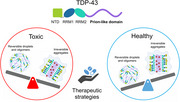

## 1 Introduction

Protein assembly has emerged as a key area to understand the balance between physiological and pathological status for different types of disorders, especially linked to neurological diseases [1, 2]. For this reason, it is very important to keep in mind that this balance is very subtle, and physiological events can become irreversible and promote pathological status [2]. In this delicate equilibrium, protein folding plays a crucial role, since approximately 44% of the human proteins were estimated to not adopt a predefined three‐dimensional structure [2, 3]. Indeed, some amino acid regions, or entire proteins, exhibit conformational flexibility, without maintaining a fixed structure over time. This is the peculiar feature of intrinsically disordered regions (IDRs), which confer structural flexibility and the ability to engage in multivalent intermolecular interactions [4]. Such interactions can be both homotypic, mediated through self‐recognition between identical proteins, or heterotypic, involving different proteins [5]. By lacking a unique structure, IDRs can adopt a continuum of interchanging conformational states, resulting in a vast plethora of possible interacting partners. For this reason, IDRs are one of the principal driving forces behind the process of liquid‐liquid phase separation (LLPS) [6]. This phenomenon is fundamental for the formation of membrane‐less organelles (MLOs), which spatiotemporally coordinate crucial cellular processes and guarantee rapid responses upon external stimuli [7].

Among the IDRs, prion‐like domains (PrLDs) are distinguished for their low complexity and their compositional resemblance to those originally found in yeast proteins [6, 8]. Unlike canonical prions, proteins bearing PrLD are not inherently pathogenic and are widely spread among eukaryotic proteins, especially regulatory proteins governing RNA metabolism and gene expression regulation [7]. Given their great involvement in LLPS, PrLDs can be considered as a conserved molecular strategy for the formation of dynamic macromolecular assemblies. However, this selective advantage comes at a cost: the intrinsic disorder of PrLD offers not only conformational plasticity, but it also confers instability and an increased tendency to aggregation [6]. Thereby, modest perturbations can tip the balance between dynamic and reversible assemblies to persistent, irreversible, solid aggregates that may induce cellular toxicity through inherent toxicity, loss‐of‐function effects, or both [6].

Protein aggregation is a multistep process in which soluble proteins progressively misfold and self‑associate into higher‑order assemblies through aberrant intermolecular interactions [9, 10, 11]. This process typically begins with nucleation, whereby a small number of misfolded molecules form an energetically unfavorable oligomeric seed that acts as a template for further recruitment of soluble monomers, followed by elongation into protofibrils and mature fibrils, often characterized by a highly ordered cross‑β‑sheet architecture [9, 10, 12]. Depending on the protein and cellular context, distinct species such as soluble oligomers, amorphous aggregates, or amyloid fibrils can accumulate, with evidence indicating that prefibrillar oligomers are often more toxic than mature fibrils in many neurodegenerative settings [13, 14]. Importantly, although LLPS can promote aggregation by concentrating aggregation‑prone species in biomolecular condensates, pathological aggregation can also occur independently of LLPS through direct misfolding and classical nucleation‑elongation mechanisms [9, 10, 15, 16].

This is exactly what happens in neurodegenerative disorders, in which the presence of insoluble pathological aggregates has become a common disease hallmark [17, 18]. Amyotrophic Lateral Sclerosis (ALS) and Frontotemporal Lobar Degeneration (FTLD), which clinically manifests in Frontotemporal Dementia (FTD), perfectly recapitulate this condition. Despite their different phenotypic manifestations, ALS and FTLD share several genetic and pathological features, including the presence of insoluble aggregates in affected neurons [19, 20]. The major component of these aggregates is TAR DNA‐binding protein 43 (TDP‐43), whose inclusions are detected in approximately 97% of all ALS cases, encompassing nearly all sporadic ALS and the majority of familial ALS cases, with notable exceptions including subsets associated with mutations in SOD1 and FUS, which instead exhibit distinct proteinaceous inclusions [17, 19, 21]. In FTLD, TDP‐43 pathology accounts for approximately 45–50% of cases, whereas other major pathological subtypes are characterized by inclusions of Tau or FUS, underscoring the molecular heterogeneity of the FTD spectrum [22]. Beyond ALS/FTD, TDP‐43 pathology is also the primary neuropathological hallmark of limbic‐predominant age‐related TDP‐43 encephalopathy (LATE), a prevalent neurodegenerative disorder of aging, and has been reported as a core pathological feature in Perry disease and in additional multisystem neurodegenerative conditions [23, 24, 25]. Nevertheless, TDP‐43 aggregates have also been detected in several other diseases, such as Alzheimer's disease (AD), Parkinson's disease (PD), Huntington's disease (HD), Dementia with Lewy Bodies (DLB), Multiple System Atrophy (MSA), Chronic Traumatic Encephalopathy (CTE), and Inclusion Body Myositis (IBM), confirming its widespread involvement in numerous neurogenerative disorders and also some that go beyond neurodegeneration [26].

**FIGURE 1:**
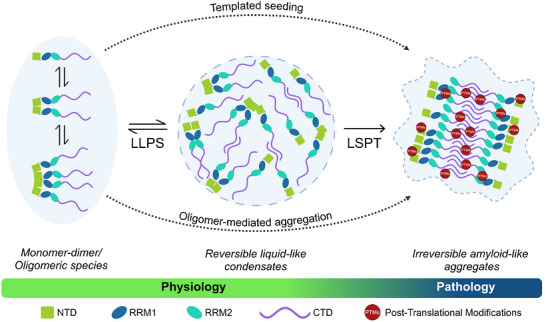
TDP‐43 polymeric continuum. Schematic representation of the different TDP‐43 assembly states observed in both physiology and pathology. This figure integrates findings from both in vitro and cellular studies and is intended as a conceptual representation rather than a direct depiction of a single experimental system. Under physiological conditions, TDP‐43 exists as an equilibrium between the monomeric and dimeric states, forming also soluble oligomeric species. Interactions between TDP‐43 N‐terminal domain (NTD) are main driver of these structures. TDP‐43 can reversibly undergo liquid–liquid phase separation (LLPS), making dynamic, liquid‐like condensates, which are primarily mediated by multivalent interactions among the NTD and the C‐terminal domain (CTD), with smaller contribution from the RNA recognition motifs (RRM1 and RRM2). By maintaining rapid molecular exchange and functional reversibility, these droplets are essential for normal TDP‐43 functionality. Perturbations of the dynamic equilibrium within phase‐separated condensates can promote a liquid‐to‐solid phase transition (LSPT), resulting in irreversible, amyloid‐like aggregates. These aggregates are insoluble and exhibit accumulation of post‐translational modifications (PTMs), including phosphorylation ubiquitination, acetylation, and PARylation. Pathological TDP‐43 assemblies can further recruit soluble TDP‐43 molecules through template‐directed misfolding, driving the formation of bigger aggregates. TDP‐43 oligomeric species were also proposed as precursors of pathological aggregates, highlighting aggregation as the end point of multiple pathological mechanisms. Importantly, LLPS can facilitate, but is not required for aggregation. Created in https://BioRender.com.

Under physiological conditions, TDP‐43 exists as a dimer or oligomer, but it can also dissociate into monomers, and can form liquid droplets via LLPS mainly mediated by its PrLD [27, 28]. However, multiple factors can perturb TDP‐43 phase separation behavior, including disease‐associated mutations, post‐translational modifications, altered availability of interactors, and chronic stress [29, 30, 31, 32, 33, 34]. These perturbations can progressively convert dynamic assemblies into irreversible, solid or gel‐like inclusions, which may also act as seeds to further promote TDP‐43 pathological aggregation [28, 29]. Therefore, the equilibrium between monomeric and dimeric TDP‐43, oligomeric species, together with reversible phase‐separated condensates, represents a continuum of interconnected states, whose relative occupancy depends on the cellular context [28, 35, 36]. In this landscape, aberrant phase transitions can drive the formation of less dynamic, solid‐like assemblies, including amyloid‐like fibrils [37, 38, 39]. While TDP‐43 aggregation can occur under physiological conditions, its dysregulation in disease contributes to neuronal toxicity [29, 40]. Together, these insights show how TDP‐43 exists as a polymeric continuum rather than in a single, fixed assembly state (Figure 1) [41, 42, 43].

## 2 The Two Sides of TDP‐43: Physiological and Pathological Roles

The *TARDBP* gene is located in the short arm of chromosome 1, specifically in the 1p36.2 locus, and encodes for TDP‐43, a well‐evolutionary conserved RNA‐binding protein (RBP) of 414 amino acids, capable of binding both UG‐rich and TG‐rich RNA/DNA sequences [44, 45]. TDP‐43 is ubiquitously expressed in all human cells and is predominantly localized within the nucleus, although it can shuttle to the cytoplasm [46]. It was originally identified as a transcription repressor of the human immunodeficiency virus type 1 (HIV‐1) through the binding to TAR (trans‐activation response) DNA element in the long terminal repeat region [47]. Nowadays, however, TDP‐43 is better known for its role as a master regulator of RNA metabolism, especially related to its splicing activity [48]. In fact, it was identified as an RNA splicing regulator by the discovery of its involvement in the exon 9 skipping of the *CFTR* transcript [49]. Shortly thereafter, CLIP‐seq experiments identified more than 6000 different human mRNAs bound by TDP‐43, highlighting its importance for the regulation of up to 30% of all coding genes [50]. Consistent with this, TDP‐43 was demonstrated to regulate every aspect of RNA metabolism, ranging from transcription, splicing, stability, localization, and translation [48].

### 2.1 Physiological Roles of TDP‐43

Regarding gene expression, TDP‐43 serves as a transcriptional regulator, acting as a regulator or repressor based on the cellular context [51]. Apart from its role in HIV‐1 transcription repression, TDP‐43 was shown to repress the transcription of the *Acrv1* gene in mouse spermatocytes by binding its promoter [52, 53]. Particularly, TDP‐43 acts as an insulator factor by disrupting the promoter interaction with the enhancer [52]. Furthermore, TDP‐43 can activate the transcription of inflammatory genes, such as *TNF* (Tumor Necrosis Factor), by cooperating with NF‐κB for the binding to its promoter [54]. Moreover, TDP‐43 alters gene expression by interacting with histone‐modifying enzymes and through the binding of long non‐coding RNAs (lncRNAs) [51].

Nevertheless, the main role of TDP‐43 is related to pre‐mRNA splicing, where it regulates a vast array of transcripts by binding preferentially to UG‐rich sequences [44]. More specifically, TDP‐43 was shown to modulate alternative splicing in all its different forms, including exon skipping/inclusion [49, 50, 55, 56], selection between mutually exclusive exons [57], intron retention [58], and regulation of both alternative splice site and alternative poly‐adenylation site usage [50, 59]. Indeed, it regulates splicing events of a myriad of important genes, such as *FUS* (Fused In Sarcoma), *SNCA* (α‐Synuclein), *APP* (Amyloid Precursor Protein), *MAPT* (Microtubule‐Associated Protein Tau), *HTT* (Huntingtin), *HNRNPA1*, *HNRNPA2B1*, *HNRNPC*, and *TARDBP* itself [29, 60]. Interestingly, most TDP‐43 binding sites reside within introns, particularly in long introns [58, 61]. In these regions, TDP‐43 has been shown to repress the inclusion of cryptic exons (CEs), which are normally excluded from the final mRNA, but are instead aberrantly included in pathological conditions, becoming a signature of TDP‐43 loss‐of‐function [62, 63, 64].

Beyond intronic regions, TDP‐43 binding sites can be found also in 3’UTR regions, thus playing a role in mRNA stability and localization. For instance, TDP‐43 stabilizes the transcripts of the human *NFL* (Neurofilament Light Chain) and *HDAC6* (Histone Deacetylase 6), increasing their half‐life through 3’UTR binding [65, 66]. Moreover, TDP‐43 was shown to increase the stability of *NOS1AP* (Nitric Oxide Synthase 1 Adaptor Protein) mRNA, although the exact mechanism is still not elucidated [67]. On the other hand, it can promote mRNA turnover, as observed for *Vegfa* (Vascular Endothelial Grow Factor A) and *Grn* (Granulin Precursor) [68].

By assembling Ribonucleoprotein (RNP) granules, TDP‐43 is also able to regulate the transport of mRNAs to specific cellular locations. In a neuronal context, this is particularly relevant, as RNP granules move bidirectionally along the microtubules to deliver specific transcripts toward the axon terminal [69].

Finally, regarding the regulation of translation, TDP‐43 has been long shown to be one of the components of stress granules (SGs) [70]. SGs are a class of MLOs, composed of both phase‐separated RBPs and RNA molecules, and represent a protective response following several stresses (oxidative, heat shock, chemical exposure, infections, etc.) [71]. SGs are reversible assemblies, as they dissolve following stress relief. Therefore, SGs act as temporary translation blockers, providing a safe storage site for stalled RNAs and RBPs. However, SGs allow translation of specific factors that are essential for stress response, including DNA repair factors, pro‐survival proteins, chaperons, as well as proteins involved in integrated stress response. Upon cellular stress, TDP‐43 colocalizes with several SGs markers (including G3BP1, TIA‐1, and TIAR), which has been proposed to indicate a protective role [70]. However, the mechanisms underlying any such protective effects of TDP‐43 recruitment to SGs remain unclear. Under physiological conditions, stress granules are highly dynamic and reversible assemblies that typically dissolve upon stress relief without progressing toward aggregation. Even so, prolonged stress or TDP‐43 mutations can disrupt the dynamicity of the condensate, resulting in irreversible pathological aggregates [72]. It is still not fully elucidated how TDP‐43 mutations can impact stress response. However, it is suggested that misfolded and aggregation‐prone TDP‐43, together with persistent stress stimuli, may cause the formation of irreversible SGs, which eventually result in pathological TDP‐43 inclusions [70, 73, 74, 75]. Nevertheless, TDP‐43 aggregation has been reported in the absence of SG formation, suggesting that it can be achieved in different ways [73, 76].

### 2.2 TDP‐43 Pathology in Neurodegenerative Diseases

The turning point in TDP‐43 research came with its discovery as the major component of insoluble aggregates accumulated in neurons of ALS and approximately half of all FTLD patients in 2006 [17, 18]. Since then, the presence of TDP‐43 inclusion has become a pathological hallmark for these diseases, making them referred to as TDP‐43 proteinopathies [77]. TDP‐43 positive aggregates can be detected in approximately 97% of ALS cases, and 45% of FTLD cases, with aberrant ubiquitination and phosphorylation in both cases [17, 18, 19]. Aggregation is preceded by the nuclear depletion of native TDP‐43, which is then accompanied by its consequent cytoplasmic accumulation. This process is assisted by the cleavage of TDP‐43 into C‐terminal fragments (CTFs), which lack the Nuclear Localization Signal (NLS) [78]. The nuclear‐to‐cytoplasmic mislocalization of TDP‐43 severely impacts its functions within the nucleus, leading to widespread deleterious effects on RNA splicing. In particular, the inclusion of CEs in the mRNA of *STMN2* (Stathmin 2), *UNC13A* (Unc‐13 Homolog A), and *HDGFL2* (Hepatoma‐derived Grow Factor Like 2) have recently become a pathological signature of TDP‐43 pathology in ALS and FTLD [62, 63, 64, 79, 80]. In addition to splicing, TDP‐43 defects have implications also in mRNA transport along the neuronal terminals and the regulation of local translation, as well as chromatin remodeling and DNA‐damage repair [78, 81, 82].

Deleterious effects of TDP‐43 inclusions may arise from the aggregation process itself. The formation of TDP‐43 amyloid‐like aggregates is the result of different predisposing factors, such as mutations in *TARDBP*, alterations in TDP‐43 expression and post‐translational modifications (PTMs) levels, and chronic stress events [74]. Nowadays, approximately 80 different mutations in *TARDBP* have been identified in ALS and FTLD patients [29, 48]. Although mutations in *TARDBP* are causative in a subset of familial ALS and FTLD cases, TDP‐43 aggregation is also widely observed in the absence of such mutations. Notably, *TARDBP* mutations account for only 10% and 20% of familiar ALS and FTLD cases respectively, indicating that TDP‐43 pathology can arise independently of genetic alterations in the protein itself [19]. Interestingly, mutations in TDP‐43 do not seem to affect a specific function and they have been shown to alter a myriad of its aspects, including the proper folding, the nuclear‐cytoplasmic shuttling, the regulation of PTMs, its RNA‐binding activity, its phase‐separation capabilities, and aggregation propensity [83, 84].

Moreover, TDP‐43 was found to aberrantly localize within the mitochondria, leading to several mitochondrial dysfunctions [85]. These include altered fission/fragmentation dynamics, increased oxidative stress, and disruption of mitochondrial‐ER connections [29]. Considering that neurons heavily rely on mitochondria for energy production, their alteration may contribute to the global TDP‐43 toxicity. In addition, the excessive presence of ubiquitin‐positive cytoplasmic inclusions affects the ubiquitin‐proteasome system (UPS), resulting in impaired proteasome‐dependent degradation and impaired autophagy procedure [86, 87]. Finally, it is important to consider that TDP‐43 exhibits prion‐like properties, meaning that its misfolding and aggregation can be propagated from cell to cell, even acting as seed for aggregation of wild‐type TDP‐43 in recipient cells [88, 89].

Given all these pieces of evidence, TDP‐43 pathology results from the combination of both loss‐of‐function (LOF) and gain‐of‐function (GOF) mechanisms, contributing together to neuronal toxicity and cell death. Quantitative studies in cellular models of ALS/FTLD have shown that nuclear TDP‑43 depletion (LOF) accounts for approximately 55–60% of toxicity, whereas cytoplasmic TDP‑43 accumulation and inclusion formation (GOF) are responsible for roughly 40–45%, depending on the assay endpoint [90, 91]. These findings indicate that the overall pathogenic impact of TDP‑43 proteinopathies arises from the co‑amplification of both LOF and GOF processes, rather than from either mechanism in isolation.

## 3 Structural Determinants of TDP‐43 Aggregation

**FIGURE 2:**
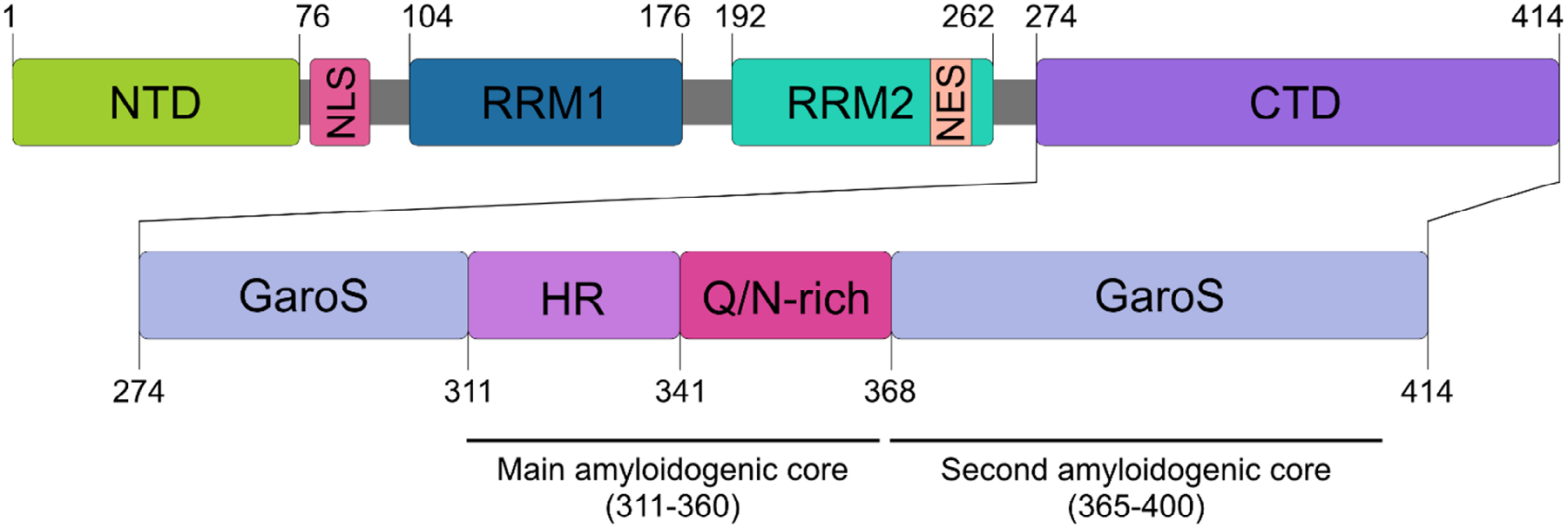
Schematic representation of TDP‐43 domains organization. TDP‐43 consists of: N‐terminal domain (NTD, 1–76), two RNA Recognition Motifs (RRM1, 104–176; RRM2, 192–262), and a C‐terminal domain (CTD, 274–414). A Nuclear Localization Signal (NLS, 82–98) is located within the N‐terminal region, while a Nuclear Export Signal (NES, 239–250) is present in the RRM2. CTD, also referred to as Prion‐like domain, is made of two regions enriched in glycine, aromatic residues, and serine (GaroS, 274–311 and 368–414) which are flanking a hydrophobic region (HR, 311–340) and a glutamine and asparagine‐enriched region (Q/N‐rich, 341–367). HR and Q/N‐rich regions together constitute the main amyloidogenic core of TDP‐43, while the C‐terminal GaroS was proposed a secondary amyloidogenic core, which is predominant during droplet maturation as observed in in vitro condensate aging studies [92].

To fully understand the molecular mechanisms of TDP‐43 phase separation and irreversible aggregation, TDP‐43 structure must be considered. TDP‐43 is composed of the typical domains present among most members of the hnRNP (heterogeneous nuclear RNP) protein family, of which TDP‐43 is a member. Accordingly, TDP‐43 basically consists of an N‐terminal domain (NTD), two RNA Recognition Motifs (RRMs) placed in tandem, and a C‐terminal domain (CTD) (Figure 2).

### 3.1 N‐Terminal Domain

The N‐terminal region of TDP‐43 contains a structured NTD (residues 1–76) and exhibits a ubiquitin‐like Dishevelled/Axin (DIX) folding, with a globular portion followed by an unstructured region [29, 93]. Within this region resides a bipartite Nuclear Localization Signal (NLS, residues 82–98), which are responsible for TDP‐43 predominant nuclear localization [29, 46]. In particular, the NLS is composed of a NLS1 (K82RK84) and NLS2 (K95VKR98), separated by a flexible linker [29]. Despite this, TDP‐43 can also enter the nucleus in an NLS‐independent manner through KPNB1 (Karyopherin Subunit Beta 1), which facilitates the interaction between TDP‐43 and FG repeats in Nups62 (Nucleoporin 62) [94].

The NTD plays a role in TDP‐43 self‐assembly, especially the first ten residues [95], as it allows the formation of dimers and oligomers in a concentration‐dependent manner [27, 96]. This strongly indicates that cellular TDP‐43 exists in a monomer‐dimer/oligomer equilibrium in healthy conditions [27, 96]. Individual monomers are assembled by head‐to‐tail interactions of the two NTDs, although a head‐to‐head interaction is also possible [29, 30, 41, 97]. Specifically, NTD was observed to form linear polymers in vitro, with both long and short chains in equilibrium simultaneously with monomers [30]. In this context, NTD acts as a recruitment platform, with its structure described as essential for proper RNA splicing regulation, phase separation, nuclear localization, enhancing DNA‐binding properties, and it has been proposed to even prevent TDP‐43 fibrillization [30, 36, 96, 98, 99]. Indeed, NTD was shown to antagonize aggregation by separating the aggregation‐prone CTD of adjacent monomers [98]. However, whether the NTD‐driven oligomerization prevent fibrillization while promoting condensate formation is still debated. Moreover, in a cellular model with inducible expression of an aggregation‐prone TDP‐43 mutant, the deletion of residues 1–75 decreased the levels of insoluble wild‐type TDP‐43, suggesting that the NTD may not be central for the aggregation, but it may help in sequestering soluble TDP‐43 into the aggregates [100]. For these reasons, the NTD can be considered an important modulator of TDP‐43 phase separation, mainly by promoting oligomerization [30]. Its alteration may therefore contribute to the formation of less dynamic or insoluble condensates, although TDP‐43 phase behavior also critically depends on CTD‐mediated interactions and RNA binding.

### 3.2 RNA Recognition Motifs

TDP‐43 has two tandem RRMs, namely RRM1 (104‐176) and RRM2 (aa 192–262), which are critical for its RNA‐binding activity, preferentially to UG‐rich RNA sequences [29, 45]. Both RRMs consist of a five‐stranded β‐sheet packed between two α‐helixes, organized in a β1‐α1‐β2‐β3‐α2‐β4‐β5 pattern [74]. Within RRM2 resides a Nuclear Export Signal (NES) (aa 239–250), which was initially proposed to mediate TDP‐43 shuttling for the nucleus to the cytoplasm [46]. However, recent findings indicate that TDP‐43 nuclear export is taking place mainly through passive diffusion [101].

Although both RRMs are functional and able to bind RNA and DNA, RRM1 shows a higher affinity for UG‐repeats, making RRM1 necessary, although not sufficient, for RNA interactions, while RRM2 modulates the sequence specificity, indicating also a synergistic binding activity [45, 102, 103]. Interestingly, TDP‐43 binds RNA in a cooperative way and in a polarized manner, meaning that RRM1 and RRM2 bind the 5’ side and 3’ side of RNA molecules respectively [104]. This property is also mediated by the short linker sequence between the two RRMs and that also contributes to TDP‐43 solubility, as expression of cooperativity‐deficient mutants in HeLa cells resulted in cytoplasmic inclusions [104].

Interestingly, RRM1 has higher affinity for ATP compared to RRM2, a feature that enhances thermodynamic stability of the domain and prevents TDP‐43 aggregation [105]. On the contrary, RRM2 contributes to TDP‐43 dimerization by increasing the thermal stability of the dimer [106]. In addition, RRM2 adopts several transient, partially folded states in native conditions [107]. A common feature of these states is the loss of elements of its secondary structures, especially of the β3, β4, and β5 tracts [107]. The hydrophobic peptides within these regions are consequently exposed to the solvent, thus manifesting their aggregation‐prone potential and ultimately resulting in fibril formation [107, 108]. Notably, RRMs have been recently linked with the aggregation process, since both RRM1 alone and in tandem with RRM2 show amyloid‐like properties and form β‐sheet enriched aggregates in vitro following heat treatment [103].

In conclusion, RRMs’ properties may contribute to the overall TDP‐43 phase separation and aggregation process, which can be also driven by TDP‐43 RNA‐binding activity per se [99, 109, 110]. However, other regions of the protein also play a critical role in modulating phase behavior. In particular, the NTD has been shown to enhance the phase separation of TDP‐43 RRMs and even drive other heterologous globular proteins [35]. Indeed, multiple proteins can bind the same RNA molecules, which serve as a multivalent scaffold for all these RBPs and thus promote intermolecular interactions and phase separation [7].

### 3.3 C‐Terminal Domain (Prion‐Like Domain)

The CTD (aa 274–414) is the key driver of TDP‐43 phase separation, due to its highly intrinsically disordered nature and the presence of an amyloidogenic core consisting in a hydrophobic region (aa 318–340) and a glutamine and asparagine‐enriched (Q/N‐rich) region (aa 341–367) (Figure 2) [84, 111]. The hydrophobic region adopts transient α‐helical conformations, which promote higher‐order oligomerization and LLPS [43, 112, 113, 114]. However, this region can undergo α‐to‐β secondary structure transitions forming β‐sheet rich filaments within TDP‐43 pathological aggregates which are Thioflavin T (ThT)‐positive, suggesting that it may initiate TDP‐43 aggregation process [115]. Supporting this, mutations that introduce charged residues in this segment (A324E or M337E) reduced the aggregation propensity due to electrostatic repulsion, while the double mutant completely prevented aggregates formation [115]. These pieces of evidence consolidate the hydrophobic region as crucial for the secondary structure transition of full‐length TDP‐43 and consequently to its overall aggregation tendency.

The hydrophobic region is followed by the Q/N‐rich region, which has high propensity to form amyloid‐like β‐sheet structures. Such structures are organized in steric zippers, which isolate the fibril core from solvent and the surrounding environment, thus enhancing the fibrillization process [111, 116]. Supporting this, an experimental model with twelve tandem repetitions of the Q/N‐rich region has been shown to be sufficient to induce highly efficient aggregation in culture cells [100, 117].

Flanking the amyloidogenic core, there are two segments that are enriched in glycine, aromatic residues and serine (GaroS), which span between aa 274–311 and aa 368–414 (Figure 2) [84, 118]. Notably, GaroS regions are formed by three “stickers”, mainly composed by Phe, Tyr, and Trp residues that are responsible for intermolecular interactions, separated by two “spacers”, which are 3–12 residues long, mainly composed by Glycines and Serines [5, 118].

Considering its unusual amino acidic composition, the CTD is also referred to as Low Complexity Domain (LCD), and it has been also termed Prion‐like domain (PrLD) because of its sequence resemblance to yeast prion proteins, such as Sup35. Along these sequences there are several intrinsically disordered regions (IDRs), which represent around 70% of the entire CTD [29, 84]. The presence of IDRs allows PrLD to adopt a spectrum of transient conformations, each available for interactions with other protein partners or with TDP‐43 itself [4, 119]. Indeed, the PrLD is key determinant for the formation of RNP granules and SGs [120, 121]. The equilibrium between the different IDR ensembles is governed by several factors, such as PTMs, shifts in TDP‐43 or its partners concentration, and mutations [5, 6, 122]. Unsurprisingly, the PrLD is the main site harboring most disease‐related mutations [29, 48]. In fact, the PrLD represents not only the main core for TDP‐43 LLPS, but also for the formation of amyloids with typical cross‐β fold [123]. This makes the PrLD the core of TDP‐43 dual nature: crucial for the regulation of LLPS in physiological conditions but also representing the pathological turn out in neurodegenerative diseases. Indeed, the PrLD is central in the so‐called liquid‐to‐solid phase transition (LSPT) of TDP‐43, converting liquid condensates into irreversible solid aggregates [5, 28].

## 4 TDP‐43 Biomolecular Condensates Formation in Physiology

### 4.1 Liquid‐Liquid Phase Separation (LLPS): Characteristics and Mechanisms

TDP‐43 forms liquid‐like condensates through a principle called LLPS. In general, LLPS is a chemical‐physical principle in which a homogenous solution of macromolecules starts to de‐mix into two coexisting liquid phases: a dense one, enriched in specific macromolecules, and a dilute one, with lower concentration of those specific components [31]. In cells, the dilute phase is not lacking of macromolecules; instead, selective enrichment within condensates occurs without altering overall crowding outside them [31]. This process is reversible, and the macromolecules concentration of the two phases can differ by up to 50‐fold, allowing local accumulation of proteins in specific cellular compartments [5]. The dense phase appears as a liquid‐droplet, with non‐Newtonian liquid properties, such as the spherical shape, the capability of undergoing fusion and fission, and internal dynamicity [19]. LLPS can involve a single component (homotypic phase separation) or at least two different components (heterotypic phase separation), as it is more likely to occur within biological systems [5]. Indeed, in cells LLPS is the driving regulating MLOs formation and its first identification took place in 2009 with the discovery of liquid‐like properties of P granules in germ cells of *Caenorhabditis elegans* [16]. Since then, LLPS was described as the principle behind the nucleolus, nuclear speckles and paraspeckles, RNA granules, SGs, and synaptic density among others [124].

LLPS occurs when the concentration of macromolecules such as proteins, DNA, RNA or poly(ADP‐ribose) polymers exceeds a critical threshold, promoting their demixing from the surrounding environment. Such macromolecules act as scaffolds (or drivers) of phase separation through multivalent interactions among themselves, forming the network that underlies condensate assembly. Client proteins are subsequently recruited into these condensates via interactions with scaffold components [28, 31]. In this context, the interactions between the macromolecules are more energetically favorable than the macromolecules‐solvent interactions, and scaffold proteins help in reducing the free energy within the droplets despite the entropic imbalance [31]. In addition, the phase separation propensity of both scaffolds and clients can be influenced by the regulator proteins, which can alter interaction affinities by inducing PTMs [28].

A crucial concept for LLPS to happen is multivalency, which can be described as the ability of a biomolecule to simultaneously engage in multiple intra‐ and inter‐molecular interactions [5]. Multivalency directly correlates with LLPS, and it can be achieved through several ways based on the involved molecules. DNA and RNA elements, folded proteins, and IDRs. The numerous consensus binding sequences along the DNA/RNA molecules make them highly multivalent and increase the liquid‐like properties of the condensates [99, 125]. Proteins with multiple folded domains can also provide high multivalency and thus act as scaffold proteins. For instance, by mixing in vitro protein containing either SH3 domains or its ligand proline‐rich motifs (PRMs), it was observed that their phase separation propensity increased progressively with the number of the involved domains [126].

In this context, the question can arise regarding how IDRs contribute to LLPS. Contrary to folded protein regions, IDRs do not adopt a single stable secondary or tertiary structure. Instead, they rather exist as a highly dynamic ensemble of conformations that interconvert rapidly and possess similar energy states [4]. This peculiar property of IDRs allows for high‐specificity/low‐affinity binding and for one‐to‐many interactions, as they can adopt several different conformations based on the interacting partners [6]. In particular, both charged and aromatic aminoacidic residues, especially the ones within the IDRs, are involved in the interaction network that drives LLPS. Since LLPS happens in the absence of covalent bonds, the entire process is mediated by electrostatic and dipole‐dipole interactions, as well as hydrogen bonds, cation‐π and π ‐π, and van der Waals interactions [6, 29].

### 4.2 Phase Separation of TDP‐43

Phase separation of TDP‐43 has been intensively studied both with purified full‐length protein or CTD fragments in vitro, and in neurons, where TDP‐43 forms condensates with a diameter of approximately 0.2‐1 µm [42, 76, 127]. This process is crucial to ensure proper TDP‐43 functionality as the expression of endogenous LLPS‐deficient TDP‐43 in mice was shown to lead to increase ribosomal assembly which can alter the rate of protein synthesis: this was followed by impaired dendrite arborization and neurological defects, even in the absence of TDP‐43 proteinopathy [128]. Moreover, LLPS is critical for TDP‐43 splicing activity, for its own autoregulation process, and for its specific binding to the UG‐repeats across the entire transcriptome [43, 99, 125, 129]. Nonetheless, it has been reported also that LLPS‐deficient TDP‐43 mutants did not affect the exon 9 skipping in the *CFTR* transcript, suggesting that LLPS may be dispensable for at least some specific TDP‐43 splicing targets and that the effects of TDP‐43 phase separation may be fundamental only for specific subsets of RNAs [130].

In any case, thanks to LLPS the TDP‐43 protein can be recruited in several phase‐separated MLOs, which are fundamental to locally increase TDP‐43 levels and thus confine/increase its activity. Apart from the already mentioned SGs [70], TDP‐43 can be detected in nuclear speckles and paraspeckles [131, 132], Cajal bodies [128], stress‐induced nuclear bodies [133], processing bodies [134], and transport granules [69, 135].

Importantly, dynamic and reversible ribonucleoprotein condensates are fundamentally distinct from irreversible aggregates, as only a subset of condensates undergoes pathological maturation, while most remain transient and dissolve upon restoration of physiological conditions.

Phase separation of TDP‐43 is mainly dependent on its NTD and CTD, with additional regulation offered by several PTMs and environmental factors [30, 35, 43]. The NTD allows initial dimerization therefore increasing TDP‐43 local concentration and facilitating the nucleation process [74]. Both the deletion of the entire NTD or two single point mutations (Y4R and E17R) abolished TDP‐43 phase separation by preventing its oligomerization [99]. In parallel to this effect by the NTD, the CTD can self‐interact through the hydrophobic patch by forming transient α‐helices within the condensate [73, 113]. Interestingly, the amino acidic context of the hydrophobic region within the CTD was proposed to discourage the formation of α‐helix, as the substitution of the G335 with any other small amino acid resulted in increased TDP‐43 helicity [43]. Therefore, the CTD was proposed as a tunable module in which precise biophysical principles can regulate both protein assembly and function.

As an IDR, the CTD of TDP‐43 follows the “stickers and spacers” model, in which stickers are designated for intra‐ or inter‐molecular interactions, while the spacers allow flexibility between the stickers, modulating the interactions number [28]. Notably, the number and the position of aromatic residues that compose the sticker regions are highly conserved and are crucial drivers of TDP‐43 phase separation through π ‐π interactions [130]. Moreover, TDP‐43 mutants with a lower number of sticker regions showed more solid condensates, with reduced internal redistribution and dynamics [130]. This suggests that TDP‐43 phase separation may be directly encoded in the conservation patterns of aromatic and hydrophobic residues within the CTD [130].

Finally, also the RRM domains of TDP‐43 can play a role for TDP‐43 LLPS, In fact, during the demixing of TDP‐43 in SGs, RRM1 was observed to partially unfold, leading to solvent exposure of Cys173 and Cys175 and thus promoting disulfide bond formation [73]. Indeed, both C173V and C175V mutants exhibited a reduced phase separation of in vitro reconstituted SGs, compared to wild‐type TDP‐43, suggesting a contribution of the RRM1 in mediating LLPS [73].

Taken together, all these observations point to the fact that LLPS of TDP‐43 can be driven by all its major domains and that it therefore represents a basic and intrinsic property of the TDP‐43 protein as a whole.

### 4.3 Regulation of TDP‐43 Phase Separation

As already mentioned, the characteristics of TDP‐43 LLPS can be finely modulated by several conditions, such as pH, temperature, salt concentration, and macromolecules concentrations [31]. Turbidometry measurements of purified recombinant TDP‐43 revealed a peak in phase separation around pH 6, with a marked decrease of turbidity at lower and higher pH values [127]. In contrast, turbidity measurements of the sole TDP‐43 LCD showed a linear relation between pH and LLPS until pH 9 [42], with decreased phase separation above pH 10 [136]. This can be explained by considering the isoelectric point (pI) of the two proteins, which is 6.02 and 9.98 for the full‐length TDP‐43 and the LCD respectively [127, 136]. These results suggest that TDP‐43 phase separation is encouraged when the pH value matches the pI of the analyzed protein, highlighting the decisive role of electrostatic repulsions during condensate assembly. Although these experiments were carried out in a cell‐free system, their physiological relevance is supported by evidence that pH alterations within the cellular environment can also affect TDP‐43 phase separation and localization [137]. Both elevated and reduced pH values compared to the physiological one lead to a modest increase in cytoplasmic TDP‐43, accompanied by increased formation of SGs [137]. These results also highlight the role played by electrostatic repulsion in the aggregation process [138].

Concerning the temperature, the highest level of TDP‐43 phase separation was obtained at 31°C, with weaker droplet formation at higher temperatures (37 and 43°C) [42, 127]. This is in line with a process of spinodal LLPS in which the system is unstable and undergoes phase separation entirely, without nucleation events. During spinodal LLPS, the demixing process can be suppressed by higher or lower temperatures when the protein concentration above the saturation concentration (Csat) [127].

As expected, the LLPS behavior of both full length TDP‐43 and its LCD can be influenced by salt concentration [42, 127]. Specifically, increasing NaCl concentrations progressively promote their phase separation, reaching complete saturation at around 150 and 200 µM NaCl respectively for the full‐length protein and the LCD [127, 136]. In this context, salts facilitate charge screening, which reduces the electrostatic repulsion between the assembled molecules [136]. Interestingly, elevated salt concentrations can promote LLPS of TDP‐43 even at pH lower than the pI by screening the net positive charge of the protein [42, 127].

Next, TDP‐43 phase separation is regulated by TDP‐43 concentration itself, as higher TDP‐43 concentrations enhance the speed and the degree of phase separation [127]. Even TDP‐43 RNA‐binding activity influences its LLPS. Specifically, FRAP experiments conducted in Zebrafish demonstrated that RNA‐binding‐deficient TDP‐43 mutants (2KQ and F4L) form liquid droplets with increased dynamics and mobility [109]. Nonetheless, despite the loss of binding to RNA, these mutants exhibit prolonged temporal interactions. This means that, even if their binding capability is reduced, when the binding occurs it lasts for longer than the wild‐type protein [109]. As a result, if we consider RNA molecules as scaffolds for TDP‐43 recruitment, the number of binding‐sites they contain will affect their multivalency and thus the LLPS process. Indeed, it is considered that RNA oligonucleotides containing 6 GU repeats (GU6) can interact with one molecule of TDP‐43 [99]. Accordingly, by increasing the number of GU repeats up to 18 (GU18), which can potentially bind three TDP‐43 molecules, bigger droplets were observed. This confirmed that the sequence and length of RNA scaffolds can promote LLPS, and affect fusion and droplet coalescence [99]. Most importantly, from an eventual RNA‐based therapeutic approach, GU18 repeats were shown to rescue the phase separation of several RNA‐binding‐deficient TDP‐43 mutants (F2L, F4L, and substitution of RRMs with GFP) [99]. Interestingly, in fact, LLPS is also promoted by RNA oligonucleotides containing TDP‐43 binding‐sites without exhibiting GU repeats (as the TDPBR sequence for the TDP‐43 autoregulation process) [99, 139].

Overall, therefore, it has been widely demonstrated that elevated RNA concentrations buffer TDP‐43 droplet formation, while low RNA/protein concentration ratios promote LLPS behavior, something that has been observed in many PrLD‐containing proteins [140]. Supporting this, cells treated with RNA oligonucleotide baits based on Clip‐34 nt, a well‐characterized TDP‐43 target sequence, showed a dose‐dependent reduction of TDP‐43 assemblies and aggregates [141]. Nevertheless, the absence of TDP‐43 NTD or CTD severely impaired LLPS even with GU30 RNA oligonucleotides, suggesting that these domains are indispensable for TDP‐43 RNA‐driven LLPS [99]. Indeed, mutations in the CTD, such as A321G and M337V, result in the formation of smaller droplets in the presence of GU‐rich RNA and when these mutants are mixed with wild‐type TDP‐43 they can disrupt the phase separation of the wild‐type protein [99].

Another level for LLPS regulation is offered by PTMs. The expression of several TDP‐43 phosphomimetic mutants resulted in a reduction in its LLPS tendency, suggesting that TDP‐43 phosphorylation can enhance its solubility [30, 142, 143]. For instance, mimicking the phosphorylation of the highly evolutionary conserved S48 with a S48E mutation resulted in a reduced number of droplet formation in vitro, with faster internal kinetics [30]. Specifically, S48E mutation was shown to disrupt the linear polymerization mediated by the NTD with a dominant negative effect, impairing also the chain‐forming interaction of wild‐type TDP‐43 [30]. Moreover, S48E mutant shows an increased inclusion of *CFTR* exon 9, therefore suggesting that NTD‐driven phase separation mediates TDP‐43 splicing function [30].

Despite this, most TDP‐43 phosphorylation sites reside within the CTD. Mass spectrometric studies in the spinal cord of ALS patients detected TDP‐43 phosphorylation in 12 out of 14 Ser residues of the CTD, which includes S373, S375, S379, S387, S389, S393, S395, S403, S404, S407, S409, and S410 [144, 145]. Phosphomimetic mutations of these residues in different combinations showed a gradual reduction of droplet formation as the number of phosphomimetic residues increased, accompanied by increased droplet fluidity and faster FRAP times [143]. Particularly, the S333D mutant displayed a shorter transient α‐helix within the CTD and this prevents the secondary structure stabilization of this region through intermolecular interactions [142]. Similarly, molecular dynamics simulations of the S375E phosphomimetic mutant revealed an increased structural instability and a greater inter‐strand distance compared to wild‐type TDP‐43 [32]. This suggests that phosphorylation induces dynamic conformations that disrupts TDP‐43 homotypic interactions, impairing LLPS and potentially modulating aggregation. However, the effects of phosphorylation appear to be context‐ and site‐dependent: while hyperphosphorylation across multiple residues tends to suppress phase separation and increase protein solubility in a charge‐dependent manner, phosphorylation at specific sites has also been reported to promote aggregation and to differentially impact on TDP‐43 splicing activity [146, 147]. Accordingly, the S375E mutant showed increased cytoplasmic localization, suggesting that phosphorylation at this site may affect TDP‐43 subcellular distribution [32]. However, this relationship is not straightforward because hypo‐phosphomimetic mutants of TDP‐43 CTD were observed to accumulate in the cytoplasm, whereas hyper‐phosphomimetic mutants showed increased nuclear localization compared to wild‐type TDP‐43 [148]. Presumably, this could be due to the introduction of additional negative charges that may increase the electrostatic repulsion between TDP‐43 molecules or interfere with the protein‐interaction profile of the CTD, thus influencing the protein localization. Nonetheless, considering that changes in TDP‐43 localization can certainly influence its aggregation propensity [149], it is reasonable to hypothesize that phosphorylation may indirectly affect TDP‐43 condensate behavior by modulating its nucleo‐cytoplasmic distribution, even though the specific mechanism underlying this link remains to be directly demonstrated.

Another PTM which greatly affects TDP‐43 phase separation is acetylation. The acetylation‐mimic K84Q mutation within the NLS impaired TDP‐43 nuclear localization, thus leading to its mislocalization [150]. Moreover, a non‐disease‐associated K136Q mutation inside the RRM1 that mimicked acetylation resulted in RNA‐binding deficiency, accompanied by reduced splicing activity [150, 151]. Consequently, K136Q resulted in aberrant phase separation, with bigger and more viscous assemblies that were associated with spherical droplets where TDP‐43 is enriched in the outer shell and depleted in the center [150, 152]. Since these droplets exhibit anisotropic properties, such as birefringence and liquid crystal‐like shell, they were called anisosomes [152]. Interestingly, acetylation may also promote other TDP‐43 PTMs, as TDP‐43 K136Q mutant showed higher phosphorylation at S409/410 and increased ubiquitination [150, 151], which are the hallmarks of TDP‐43 inclusions in ALS patients [17, 153]. Taking together these pieces of evidence, acetylation on lysine residues reduces the net charge of the protein, therefore promoting phase separation. Confirming the critical role of K136 in TDP‐43 phase behavior, the lysine‐deficient K136R mutant was shown to promote the nucleation propensity and condensate dynamics within the nucleus [110].

SUMOylation is another important regulator of TDP‐43 phase behavior, although its precise effects remain unsolved and presumably depend on the SUMO paralog involved and the modified residue(s). Already proposed as a SUMOylation site candidate, K136 was described to undergo SUMO1 conjugation, and K136 mutation or deSUMOylation altered TDP‐43 nuclear localization, splicing activity (particularly exon skipping), and stress granule recruitment [154]. More recent studies have shown stress‐induced SUMO2/3 conjugation at multiple lysine residues, even within the C‐terminal domain [155, 156]. SUMO2/3 conjugation has been associated with increased solubility, enhanced turnover, and protection from stress‐induced aggregation, supporting a predominantly protective role in cellular models of ALS/FTD [155, 156, 157]. Overall, rather than directly inhibiting or promoting LLPS, SUMOylation is best interpreted as a context‐dependent regulator of TDP‐43 compartmentalization and aggregation propensity.

Importantly, PARylation can also modulate TDP‐43 phase behavior, as non‐covalent binding of poly(ADP‐ribose) (PAR) chains promotes TDP‐43 phase separation and recruitment into stress granules through multivalent interactions, whereas sustained PAR signaling under chronic stress conditions can favor pathological aggregation [158]. In line with this, poly(ADP‐ribose) polymerase (PARP) activation has been associated with cytoplasmic accumulation of TDP‐43, while pharmacological inhibition of PARP or Tankyrases mitigates downstream aggregation without fully preventing stress granule assembly, highlighting a separation between condensate formation and pathological maturation [158, 159].

Overall, the impact of PTMs on TDP‐43 LLPS is strongly influenced by cellular context, including acute versus chronic stress conditions, subcellular localization, and extensive crosstalk among different modifications, which together determine whether phase separation remains reversible or progresses toward pathological aggregation [34].

## 5 From Physiological Assembly to Pathological Aggregation: The Contribution of the PrLD

While the IDR within the CTD of TDP‐43 confers important functional advantages, such as multivalent interactions that give rise to LLPS and the formation of MLOs, it also introduces an inherent vulnerability. Subtle alterations in the cytoplasmic environment, prolonged cellular stress, disease‐associated mutations or PTMs of the IDR itself can increase the aggregation propensity of this region, promoting the maturation of TDP‐43 liquid droplets into irreversible solid assemblies, consequently triggering TDP‐43 proteinopathy. The intrinsic susceptibility of the IDR is encoded in its primary sequence, which shares compositional and functional similarities with prion proteins originally described in yeast [160]. For this reason, the IDR of TDP‐43, as well as that of several other RBPs, has been termed Prion‐like, underscoring the pathological potential of this region through protein misfolding, self‐templating aggregation, and spreading of the disease. Indeed, a combination of prion‐like compositional bias (PLAAC) and sequential amyloid propensity (pWALTZ) analysis predicted the presence of 242 polypeptides containing a PrLD within the human proteome [8]. Apart from TDP‐43, the molecular functions of these polypeptides are related to DNA/RNA‐binding, transcriptional regulation, mRNA processing, being present in other proteins implicated in neurodegenerative processes such as FUS, hnRNPA1, hnRNPA2/B1, EWS [8].

Therefore, the PrLD embodies the dual nature of TDP‐43: the same structural feature that enables dynamic and reversible phase separation under physiological conditions can also drive a pathological transition toward stable and self‐sustaining aggregated states following perturbations of the delicate homeostatic equilibrium. This double‐faced role of the PrLD justifies the high selective pressure to preserve these sequences during evolution despite their intrinsic aggregation propensity.

To better understand the prion‐like behavior of misfolded proteins in neurodegenerative diseases and their parallelism with canonical prion proteins, prion diseases and prion proteins will be briefly introduced.

### 5.1 Prion Diseases

The term “prion” was originally coined by Stanley Prusiner to describe the proteinaceous infectious particle responsible for the scrapie disease [161]. Prion proteins (PrPs) represent a unique class of infectious agents whose pathological potential relies exclusively on protein conformation, in the complete absence of any nucleic acid. This “protein only” hypothesis was confirmed with the observation that PrPs can misfold into pathological conformations which ultimately result in the formation of neurotoxic aggregates [161]. These findings proved that structural information can be transferred from protein‐to‐protein without the need for nucleic acid, thus challenging the central dogma of molecular biology.

Prion diseases, or transmissible spongiform encephalopathy (TSE), are a class of fatal neurodegenerative disorders characterized by long incubation periods that reflect the progressive accumulation of misfolded proteins. In contrast, once the symptoms emerge, the disease progression is extremely fast and aggressive, consistent with the exponential spread of the misfolded protein leading to neuronal damage. During pathogenesis, the host normally folded prion protein (cellular prion protein, PrP^C^) undergoes misfolding acquiring a pathological conformation, referred to as scrapie prion protein (PrP^Sc^) for its first discovery in scrapie‐affected goats and sheep [161, 162]. This process, named template‐assisted conversion or seeding model, can be divided into three steps:

‐ nucleation, in which spontaneous misfolding events, genetic predisposition or transmission of PrP^Sc^ allow the conversion of PrP^C^ into PrP^Sc^ [161]. The altered secondary and tertiary structure of the newly formed PrP^Sc^ can act as seed or nucleation particle for the conversion of other PrP^C^ molecules through the process of template refolding.
‐ elongation, consisting of a conversion chain reaction which exponentially increase the accumulation of PrP^Sc^.
‐ fragmentation, in which the aggregate fibrils break into smaller pieces, allowing the propagation of PrP^Sc^ along other cells. This results in the formation of amyloid plaques in the affected neurons, leading to neurodegeneration by prion disease. Notably, a peculiar property of prion is the transmission to other permissive individuals, which inevitably requires the presence of PrP^C^ in the recipient organism for its conversion into PrP^Sc^ [163].

PrPs are evolutionary conserved proteins found across a wide range of organisms, including yeasts, bacteria, plants, and mammals [164]. In humans, normal PrP^C^ is a monomeric cell‐surface glycoprotein of 27–30 kDa, encoded by the *PRNP* gene and predominantly expressed in the CNS [164, 165]. Structurally, mature PrP^C^ is organized in an intrinsically disordered N‐terminal domain and a globular C‐terminal one, which consists of three α‐helices and two small antiparallel β‐sheets. The C‐terminal domain undergoes α‐to‐β secondary structure transition during the pathological conversion to PrP^Sc^, leading to β‐sheet–rich, aggregation‐prone species that form protease‐resistant amyloid fibrils. Recent cryo‐EM studies supported the parallel in‐register intermolecular β‐sheet (PIRIBS) model for the structure of prion fibrils. According to this model, the β‐strands of each molecule are oriented in the same direction, and multiple PrP^Sc^ stack side by side to form an extended β‐sheet structure which constitutes the fibril core [166, 167].

Notwithstanding their high structural conservation during evolution, prions exist as multiple distinct strains, which are characterized by different incubation times, resistance to proteases, disease manifestation, and structure of the misfolded protein [168, 169, 170]. The strain properties are intrinsically encoded in the various PrP^Sc^ structures, which are indeed maintained after serial passages across different individuals both in vitro and in vivo [171, 172].

### 5.2 The Prion‐Like Concept in Neurodegenerative Diseases

The observation that some pathological features of neurodegenerative diseases, such as the anatomic spread, phenotypic heterogeneity and the persistence of misfolded proteins, could not be fully explained by a protein aggregation model led to the extension of the prion concept. For this reason, the “prion‐like” term was introduced to describe IDR or LCD‐containing proteins that can initiate a template‐mediated misfolding of normally structured proteins in a self‐perpetuated manner and that can propagate along interconnected neuronal pathways [88, 173]. Unlike bona fide prions, however, there is no evidence of inter‐organism transmissibility.

For this reason, the use of the term “prion‐like” to neurodegenerative proteinopathies has been debated, claiming that it overestimates the similarities with bona fide prion disease. Some authors suggested that it should be reserved for conditions involving PrP and extended to other disorders only when PrP is involved, to avoid conceptual parallelism with prion diseases [174]. An alternative view is the selective vulnerability hypothesis, which attributes disease progression to intrinsic differences in neuronal susceptibility to proteotoxic stress; these models are not mutually exclusive and may coexist [173, 175].

Apart from TDP‐43, several proteins involved in neurodegeneration are currently defined as prion‐like. Regarding Alzheimer's disease (AD), β‐amyloid represents one of the best‐characterized examples of non‐PrP proteins undergoing prion‐like seeded aggregation and self‐propagation in vivo, thereby extending the prion paradigm to broader neurodegenerative proteinopathies [173, 176, 177]. Both β‐amyloid and Tau deposits were demonstrated to act as seeds, form different strains, and to spread to connected brain regions [178]. Moreover, human transmission of β‐amyloid pathology has been reported by contaminated surgical procedures and transplants of cadaver‐derived pituitary hormone extracts, further confirming the similarities to prions [179]. Importantly, Tau diffusion was observed both in the presence and in the absence of endogenous wild‐type Tau, suggesting that the endogenous protein is dispensable for the disease propagation, which is in contrast with the prion concept [180].

In Parkinson's disease (PD), α‐synuclein displays typical prion features such as seeding and propagation [181, 182]. Engraftment of fetal ventral mesencephalic tissue into the striatum of PD patients resulted in the appearance of α‐synuclein and ubiquitin‐positive Lewis bodies in the healthy grafted dopaminergic neurons, providing an in vivo evidence of the prion‐like cell‐to‐cell propagation of α‐synuclein [183].

Regarding ALS, TDP‐43 is not the only pathological protein exhibiting prion‐like behavior. Up to 20% of fALS cases are caused by mutation in copper‐zinc superoxide dismutase 1 (*SOD1*) gene, which results in a non‐functional and toxic misfolded protein [184, 185]. Interestingly, SOD1 lacks a PrLD domain, based on sequence homology. However, fALS causative SOD1 mutations make the hydrophobic core of the protein more exposed [186], resulting in an increased aggregation propensity and fibrillization in mouse models [187]. Moreover, considering that SOD1 can be secreted and released in the cerebrospinal fluid (CSF), mutant SOD1 aggregates were shown to cross the cellular membrane in recipient cells, where they could act as seeds and catalyze the conversion of normal SOD1 into the misfolded form [188, 189, 190, 191]. Also, FUS exhibits prion‐like properties driven by its N‐terminal PrLD, with multivalent π–π and cation–π interactions regulating phase separation [192]. Pathogenic mutations affecting these interaction‐prone regions affect the liquid state of FUS assemblies, resulting in toxic solid aggregates [122].

### 5.3 The PrLD of TDP‐43 as a Driver of Liquid‐to‐Solid Transitions

**FIGURE 3:**
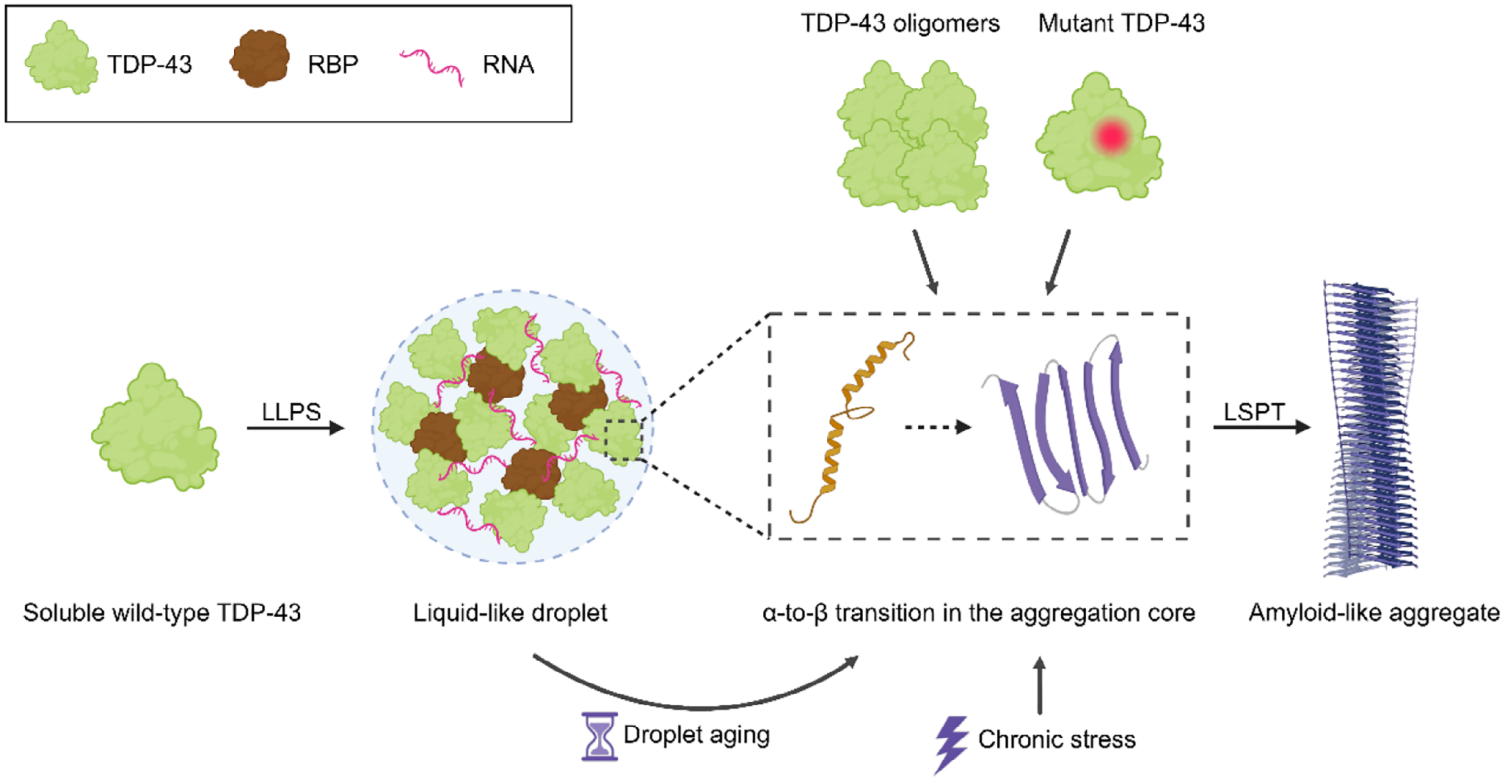
Molecular mechanisms for TDP‐43 pathological phase transition into amyloid‐like aggregates. Soluble wild‐type TDP‐43 engages in multivalent interactions with RNA and other RNA‐binding proteins (RBPs), giving rise to liquid‐like droplets by liquid–liquid phase separation (LLPS). Within these condensates, the aggregation core within the C‐terminal domain (CTD) adopt transient α‐helical structures which provide the dynamicity and reversibility of the droplets. However, persistent phase‐separated droplets undergo aging process, in which they become less reversible and dynamic over time. In this process, the amyloidogenic core undergoes α‐to‐β transition, converting helical structures into β‐sheets. This process is at the basis of the liquid‐solid phase transition, resulting in the formation of irreversible TDP‐43 amyloid fibrils. Other mechanisms promote the α‐to‐β transition, including the prolonged stress conditions (which converts stress granules into pathological aggregates), mutations in TDP‐43 (especially in the CTD), and the presence of pathological TDP‐43 oligomeric species. Created in https://BioRender.com.

While phase separation is indispensable for TDP‐43 physiological functions, aberrant phase transition can turn its reversible and dynamic assemblies into irreversible, amyloid‐like aggregates. This event is largely mediated by the PrLD, in which slight perturbations (such as chronic stress, mutations, alterations in gene expression, PTMs) can trigger maturation of TDP‐43 droplets and the liquid‐to‐solid transition of TDP‐43. Specifically, it is currently though that this is mediated by a α‐to‐β transition within the secondary structure of the amyloidogenic core, which progressively shifts from a predominant helical structure observed in physiological conditions to parallel β‐sheets typical of amyloid‐like structures [73, 113]. It is also now very clear that TDP‐43 aggregation occurs faster under phase separation conditions, underscoring the relevance of LLPS as a kinetic favorable process that initially allows TDP‐43 nucleation, but then, if persistent, can result in solid aggregates [42, 193]. Therefore, the LLPS of PrLD may represent one mechanism that facilitates the formation of pathological inclusions, acting as a structural α‐β switch which favors LLPS and progressively promotes fibrillization [42, 193]. In the following paragraphs this process will be elucidated and a graphical representation is reported in Figure 3.

#### 5.3.1 TDP‐43 Aggregates and Its Amyloidogenic Core

As already mentioned, TDP‐43 aggregation follows a nucleation‐dependent multistep process in which conformational rearrangements within the PrLD promote the formation of aggregation‐prone oligomeric intermediates that subsequently mature into β‐sheet‐rich fibrillar assemblies [9, 10]. In the context of TDP‐43, this process is strongly influenced by the intrinsic amyloidogenicity of the PrLD and by factors that shift the equilibrium from reversible condensates or oligomers toward irreversible aggregation [12, 15]. The abnormal accumulation of misfolded proteins is a hallmark of neurodegenerative diseases. Pathological TDP‐43 end‐stage aggregates consist of detergent‐insoluble amyloid‐like fibrils, with diameters of 10–15 nm [19]. Cryo‐EM analysis of TDP‐43 aggregates deriving from ALS patients with FTLD‐TDP type B pathology revealed that TDP‐43 fibrils mainly consist of single protofilaments arranged in a right‐handed helical twist [194]. The highly ordered filament core is composed by the PrLD which adopts a “double spiral‐shaped” fold, where the Gly‐rich and the Q/N‐rich regions flank and wrap around a central hydrophobic core in a spiral way [194]. This architecture allows tight turns and short β‐strands, thus avoiding extended packing of β‐sheet structures, which is typical of amyloid aggregates, although TDP‐43 filaments are amyloid‐like [194]. Aggregates found in prefrontal cortex of FTLD‐TDP type A patients showed similar characteristics, but with a chevron‐like fold of the filament core [39]. This shape is defined by the presence of a kinked β‐sheet formed between residues 321–331 which represents the fourth layer of the structure and forms steric zippers with the neighboring β‐sheets [39]. Nevertheless, the helical architecture of the fibrils is largely determined by the PrLD, since in vitro generated aggregates formed by PrLD (either entirely or specific segments) display a helical protofilament, although left‐handed twisted and with structural differences from patient‐derived fibrils [195, 196]. Importantly, cryo‐EM analysis of fibrils formed by the full TDP‐43 low‐complexity domain further demonstrated that this region alone is sufficient to generate ordered amyloid assemblies and revealed additional polymorphic conformations of the fibril core, reinforcing the intrinsic structural plasticity of the TDP‐43 PrLD/LCD [195, 197]. This could explain why TDP‐43 filaments can differ between different TDP‐43 pathologies, underlying the presence of different pathological TDP‐43 species with different structures, termed “strains”. Moreover, cryo‐EM studies of the filaments from brains of FTLD‐TDP type C revealed the presence of heteromeric amyloid filaments composed of TDP‑43 and annexin A11 (ANXA11), with a left‐handed helical twist [198]. The core fold is formed by TDP‑43 residues G282/G284–N345 and ANXA11 residues L39–Y74 (within its LCD), which assemble into a parallel in‑register cross‑β structure stabilized by a central hydrophobic interface [198]. This evidence highlighted for the first time that amyloid filaments in FTLD‐TDP patients could be heteromeric and partially explain why also mutations in ANXA11 LCD could be pathogenic [198].

At the molecular level, the main amyloidogenic core spans residues 311–360, containing the hydrophobic patch and the Q/N‐rich region, which adopts a dominant cross β‐structure within the aggregates [199]. Specifically, region 341–366 acquires a parallel β‐turn conformation, which is stabilized by the hyper cooperative H‐bonds mediated by glutamine and asparagine residues [38, 116]. Moreover, thanks to the absence of a side chain, the glycine residues present in this segment create a pocket that helps efficient intermolecular packaging of the amyloid fibril [116]. Overall, solid‐state NMR (SSNMR) studies revealed that the amyloidogenic core is composed of 5 β‐strands, the predominant of which are contributed by the hydrophobic region [115, 199]. Under physiological conditions, the structure of the hydrophobic region is in a dynamic equilibrium between α‐helical, β‐sheets and disordered conformation (Figure 3). This evidence supports the importance of the progressive conversion of α‐helices into β‐sheet conformations in the aggregation core region during the phase transition [199, 200]. Several amino acidic residues have been identified as crucial for this transition and the stabilization of the amyloid structure, including F316, I318, A325, A326, Q327, A329, S332, and W334 [199]. Particularly, the interaction between the indole group of W334 and the side chain of Q343 may be crucial to initiate the misfolding of TDP‐43, as an artificial W334G mutation was shown to markedly prevent TDP‐43 phase separation [199].

Under physiological conditions, this region is partially shielded by establishing transient interactions and by the flanking regions, preventing the structural shift toward β‐structure conformations. However, perturbation of this balance can transiently expose the amyloidogenic core, promoting intermolecular β‐sheet interactions, thus leading to the liquid‐to‐solid transition of TDP‐43. As aggregation progresses, the region is buried again within the assemblies, becoming the structural core of the amyloid fibrils. Together with sporadic presence of non‐amyloid fibrils, this is the reason why, on some occasions, TDP‐43 aggregates failed to be stained by to amyloid‐specific dyes such as ThS/ThT (Thioflavin S/T) and CR (Congo Red).

#### 5.3.2 Droplet Aging

Multiple factors and mechanisms have been proposed as crucial drivers of this pathologic phase transition, which are not mutually exclusive and likely converge to promote the formation of aberrant aggregates. Considering the intrinsic nature of the PrLD, persistent TDP‐43 droplets are destined to become less dynamic and reversible over time. This phenomenon, termed droplet aging, is characterized by a time‐dependent maturation of initial liquid assemblies into increasingly rigid, solid, or gel‐like aggregates [200]. This spontaneous process, supported by several in vitro experiments, arises from stabilization of aberrant intermolecular interactions within the droplets, eventually leading to reduced mobility and irreversibility in line with the pathological aggregates found in patients. Indeed, Raman spectroscopy studies conducted on purified TDP‐43 mutant depleted of the 3 key tryptophane residues to improve its solubility (W334F/W385F/W412F, referred to as W_free_) showed aberrant phase‐separated droplets with increased β‐sheet content already after 4 h of LLPS initiation [200]. This came with a reduction in the disordered fraction, without significant alterations in the α‐helical content, and with loss of water content, a characteristic of amyloids formation [200]. After aging for 24 h, droplets became solid, as indicated by the absence of fluorescence recovery in FRAP experiments [200].

Notably, a SSNMR study identified a second amyloidogenic core in the region 365–400, which appears to represent the dominant structural core in aged TDP‐43 amyloid fibrils [92]. Specifically, while the “principal” amyloidogenic core within residues 311–360 initiates and promotes the aggregation process being thermodynamically favored, the second one is the most stable in matured droplets, although energetically discouraged [92]. However, this second C‐terminal region has so far been described in vitro in studies of phase‐separated TDP‐43 low‐complexity domains undergoing droplet aging, and is not consistently resolved in current patient‐derived cryo‐EM fibril structures.

Finally, complementary Raman spectroscopy and atomic force microscopy (AFM) studies demonstrated that fibrillary species can also arise in the bulk phase independently of LLPS, suggesting that TDP‐43 aggregation could be heterogenous. In this context, aggregates form predominantly upon droplet maturation, followed by fibrillization of soluble monomers, underscoring the multifaced nature of TDP‐43 pathology [42, 200].

#### 5.3.3 Are SGs Precursors to TDP‐43 Aggregates?

TDP‐43 undergoes LLPS to form dynamic condensates that can mature over time and, under permissive conditions, progress toward less dynamic or solid‐like states. This process is not inevitable, but depends on factors such as protein concentration, RNA binding, post‐translational modifications, disease‐associated mutations, and stress conditions. Indeed, considering TDP‐43 involvement in SGs assembly, it has been widely proposed that pathological aggregates principally derive from SGs upon perpetuated or chronic stress stimuli. Supporting this, the induction of specifically chronic oxidative stress in ALS iPSC‐derived neurons resulted in the recruitment of TDP‐43 within SGs followed by the formation of cytoplasmic aggregates containing phosphorylated TDP‐43, with a consequent loss of TDP‐43 splicing functions [34, 201]. In this context, the PrLD appears to play once again a pivotal role in the maturation of SGs in insoluble aggregates. Indeed, α‐to‐β transitions within the amyloidogenic core were demonstrated to contribute to the liquid‐to‐solid conversion of SGs, helped also by disulfide bonds occurring in the RRM1 [73]. Importantly, this model does not imply that SGs represent an obligatory intermediate in TDP‐43 aggregation, but rather one of several possible pathways whose contribution may depend on the cellular context and stress conditions.

However, it is important to note that TDP‐43 aggregation independently from SGs formation has also been reported. Specifically, it has been observed that SGs and pathological TDP‐43 inclusions have different components and morphologies. While SGs maintain a spherical shape, TDP‐43 aggregates show an amorphous or filamentous morphology [202]. Moreover, colocalization between TDP‐43 and *de novo* produced SGs was observed only at early stages, with reduction as the aggregates mature [202]. Consistently, these observations support the existence of SG‐independent aggregation routes and argue against a strictly linear progression from LLPS or SGs to fibrillar inclusions. Nevertheless, SGs may also indirectly contribute to TDP‐43 pathology by sequestering HDAC6, thus increasing the levels of acetylated TDP‐43 which in turn lead to accumulation of insoluble phosphorylated and acetylated TDP‐43 [151, 202, 203].

#### 5.3.4 Oligomers as an Intermediate Step for Aggregation

Another still unresolved question in TDP‐43 aggregation is represented by the existence of TDP‐43 oligomers in the early stages of the aggregation process. Oligomers are small, soluble, and often metastable aggregates formed during the initial stages of protein misfolding and aggregation [29, 204]. In some cases, they have been shown to act as crucial, often obligatory, intermediates that bridge the gap between individual monomers and larger, insoluble fibrils. The observation of possible pathological TDP‐43 oligomerization came from the analysis of the brain of mice models and in the hippocampus, frontal cortex and anterior orbital gyrus of ALS/FTLD patients [205, 206]. TDP‐43 PrLD can indeed form reversible oligomers with β‐sheet structure, shielding the tryptophane residues from interacting with the solvent [207]. TDP‐43 oligomers showed immunoreactivity with an anti‐amyloid oligomer antibody specific for amyloid β (A11), further suggesting a similarity between the two pathological proteins [206]. Transmission electron microscopy (TEM) and AFM studies revealed how TDP‐43 oligomeric species can be heterogenous, comprising both spheroid and ring‐shaped structures of approximately 40–60 nm [206]. Although TDP‐43 oligomeric complexes are resistant to detergents, their molecular weight roughly corresponds to tetramers which are independent from disulfide bonds, making them biochemically and structurally different from TDP‐43 aggregates [208]. Therefore, oligomers can serve as seeds for the formation of pathological aggregates.

#### 5.3.5 The Contribution of ALS‐Linked Mutations in TDP‐43 Aggregation

ALS‐linked mutations in *TARDBP* serve as strong modifiers of TDP‐43 phase behavior by increasing its aggregation propensity, enhancing the mislocalization or the protein stability, affecting the interaction with binding partners, or promoting gain of toxic functions. Consistently, out of the 80 different disease‐associated mutations in *TARDBP*, approximately 85% are clustered in the PrLD and generally act by remodeling the energetic landscape of the domain in favor of irreversible amyloid‐like aggregates [33, 207]. Rather than inducing *de novo* aggregation, these mutations widely accelerate the pathological continuum from physiological, transient condensates toward kinetically trapped and irreversible assemblies. Mutations such as A315T, A315E, A321G, Q331K, G335D, M337V, R361T, and N390D were shown to enhance TDP‐43 aggregation [29, 48, 74]. Specifically, A321G, M337V, and Q331K mutant possess a strongly destabilized helical structure within the hydrophobic region, which disrupts TDP‐43 phase separation and accelerates its aggregation in vitro, leading to amyloid fibrilization and seeding capability [113, 207, 208, 209]. Increased aggregation can affect TDP‐43 functionality, as TDP‐43 M337V knock‐in mice exhibited splicing deregulation compatible with toxic gain‐of‐function, although the absence of neurodegeneration [210]. TDP‐43 Q331K transgenic mice instead showed an altered autoregulation mechanism, followed by a loss of splicing function for a specific subset of target pre‐mRNA (such as *Kcnip2* and *Abhd14a*), while a reinforcement of the splicing function for other targets [211, 212]. Interestingly, this mouse model shows motor dysfunction and muscular atrophy even without the presence of cytoplasmic TDP‐43 inclusions [213]. In a region next to position 331, the G335D mutant was observed to promote TDP‐43 aggregation in vitro and in cellular models, accompanied by loss of TDP‐43 splicing activity, considering that this mutation destabilizes the loop in the transient helix‐loop‐helix inside the hydrophobic region [214]. Conversely, although the Q343R mutation destabilized the second helix, it decreased TDP‐43 aggregation, keeping the splicing activity intact [214].

Overall, therefore, ALS‐linked *TARDBP* mutations strongly impair the regulatory constraints governing TDP‐43 self‐association in several ways, but each mutation has its own peculiarities, and it is not easy to find a common unifying theme. Further work should certainly be focused on investigating this topic by performing experiments that analyze many mutations in parallel to allow a better comparison between them.

### 5.4 Prion‐Like Spread of TDP‐43 Pathology

Although aberrant phase transition may explain the key aspects of TDP‐43 pathology within individual cells, it is the prion‐like behavior of TDP‐43 that could eventually support the spatiotemporal progression of the pathology observed in ALS and FTLD patients. Indeed, mounting evidence indicates that TDP‐43 pathological aggregates can be transferred from one cell to another in a prion‐like manner, and they can act as seeds, triggering the aggregation of the recipient cell endogenous TDP‐43.

#### 5.4.1 Template‐Directed Misfolding and Seeding

Consistently with this hypothesis, both in vitro generated TDP‐43 fibrils and patient‐derived insoluble aggregates have been shown to be efficiently uptaken in cellular models, patient‐derived cerebral organoids, and in mouse models following their in vivo injection [88, 215, 216, 217, 218, 219, 220, 221, 222]. Interestingly, TDP‐43 aggregates can also be released by pathological cells and then uptaken by the neighbor cells, providing evidence for prion‐like propagation [89]. Once in the receiver cell, these aggregates could serve as seeds to promote a template‐directed aggregation of endogenous TDP‐43, resulting in insoluble condensates that resemble the hallmarks of TDP‐43 proteinopathies, such as nuclear clearance followed by cytoplasmic mislocalization, aberrant phosphorylation especially in S409/S410, ubiquitination, presence of CTFs and p62, which usually colocalize with TDP‐43 inclusions in ALS/FTD brains [89, 215, 216, 217, 220, 222, 223]. Notably, phosphorylation appears to be sequential from the N‐terminal toward the C‐terminal, since phosphorylation at S403/S404 was demonstrated to precede and to be necessary for phosphorylation at S409/S410 [220]. For the seeding to occur, it is fundamental that the amyloidogenic core is exposed from the fuzzy coat where it is usually buried. In fact, protease K (PK) treatment greatly increased the uptake and seeding capability of both in vitro generated and patient‐derived TDP‐43 aggregates by making the fibril core exposed [196].

In addition to this evidence, the templated fashion of TDP‐43 conversion into pathological aggregates is supported by observations showing that amyloid fibers generated from specific peptides within the PrLD can act as a seed only when the corresponding region is present in the recruited TDP‐43 protein [217]. Indeed, the nuclear clearance caused by the seeding of the neo aggregates can in turn enhance the seeding process itself and promote TDP‐43 loss‐of‐function, reported as increased cryptic exon inclusion events, alteration of MAPK, FoxO, NF‐κB pathways and p53 signaling [215, 216]. Notably, intracerebroventricular injections of ALS patient‐derived CSF in transgenic mice resulted in cognitive impairment, motor dysfunctions, weight loss, motor neuron death, neurofilament dysregulation, and denervation of neuromuscular junctions [224]. Moreover, ALS‐patient derived extracts caused astrogliosis in patient‐derived cerebral organoids, together with apoptosis and DBS induction [222].

#### 5.4.2 TDP‐43 Strains

Confirming the similarity to prions, several TDP‐43 strains have been putatively identified. Indeed, distinct structures and morphologies were observed in TDP‐43 aggregates in insoluble fractions of brain extracts derived from various subtypes of FTLD‐TDP patients [218]. The aggregates were differentially sensitive to PK digestion, suggesting different folding of the amyloidogenic core that reflects the difference in the seeding properties [218, 219]. Even the neo aggregates induced by the treatment with patient‐derived extracts show distinct properties. While FTLD‐TDP‐A neo aggregates are big, spherical and densely packed with TDP‐43 fibrils, FTLD‐TDP‐C neo aggregates appear as multiple smaller condensates, less dense in fibrils and with amorphous structure. On the other hand, FTLD‐TDP‐E neo aggregates showed a skein‐like morphology [219, 220]. These features strikingly resemble the ones of the original aggregates in the seeds of the corresponding FTLD‐TDP subtype, supporting the concept of different TDP‐43 strains with different seeding capability and spreading patterns [219, 220]. Indeed, TDP‐43 aggregates were shown to be intercellular transmitted to anatomically connected regions, preserving the conformation of the aggregates across the various brain regions [196, 219]. Therefore, each strain exhibited a specific spreading pattern along interconnected neuronal networks, with different capabilities of forming *de novo* phosphorylated aggregates and cellular localization [219]. In addition, aggregates generated in vitro during a second round of seeding could recapitulate the properties of the aggregates resulting from the first round, as observed for PrP^Sc^ [196].

#### 5.4.3 TDP‐43 Prion‐Like Propagation and Cross‐Seeding

Another question that has been investigated so far is represented by how seeds can propagate. At present, TDP‐43 aggregates have been shown to be propagated through several mechanisms:

‐ vesicle‐associated release. TDP‐43 can be packed inside microvesicles and exosomes, which promote both the release and the uptake, reflected by higher toxicity in the receiver primary neurons compared to TDP‐43 free packaged vesicles [221]. Supporting this spreading route, TDP‐43 was detected in exosomes released by Neuro2A cells, and in extracellular vesicles from sALS lymphoblasts [225]. Microvesicles and exosomes are suggested to enter the receiver cell by endocytosis or micropinocytosis [226, 227]. However, vesicles also mediate trans‐synaptic transfer, as TDP‐43 aggregates in the soma of donor neurons were observed to move anterogradely toward the axon terminal, transmitted to receiver cells, where they can be transported to the soma by retrogradely transport [221]. This evidence underlies the capability of TDP‐43 aggregates to move between the neuronal soma and the axon terminals, thus allowing long‐range propagation of the pathology.
‐ direct cell‐to‐cell transfer. Recently, it was reported that conditioned media from sALS lymphoblasts could induce not only TDP‐43 mislocalization, phosphorylation, and fragmentation to healthy cells, but it could also induce the formation of tunneling nanotubes (TNTs) [225]. TNTs are thin F‐actin‐based protrusions that establish direct physical connection between neighboring cells, creating cytoplasmic continuity and enabling transfer of ions, single molecules, organelles and even protein aggregates [228]. TDP‐43 appears to exploit TNTs for its intercellular spreading based on the observation that it was detected within TNTs‐like structures in cultured cells upon treatment with ALS‐FTD CSF samples [229]. This feature is shared with prion proteins, as their propagation is also associated with TNTs [230].
‐ Non‐vesicular release in the extracellular environment. This could be mediated by passive leakage during cell stress or membrane damage or following cell death [89].

It is worth mentioning that a membrane‐interacting domain has been identified between TDP‐43 residues 311–343, which adopts a Ω‐loop‐helix structure in membranous environments [207, 231]. Consistently, TDP‐43 can bind lipid monolayers and bilayers, and prolonged exposure can lead to pore formation and membrane damage, potentially contributing to membrane fragmentation and facilitating TDP‐43 release [232].

Finally, increasing evidence supports cross‐talk between TDP‐43 and other aggregation‐prone proteins across neurodegenerative diseases. TDP‐43 can exacerbate Tau pathology, potentially by stabilizing phosphorylated Tau species and promoting their aggregation [233], while mutant huntingtin promotes both aggregation and phosphorylation of TDP‐43, suggesting a bidirectional pathological reinforcement between these proteins [234]. Moreover, α‐Synuclein has been reported to colocalize with TDP‐43 PrLD‐containing condensates, where it may act as a Pickering‐like stabilizing agent [235], and the cellular prion protein may facilitate the uptake of TDP‐43 fibrils, linking its pathology to prion‐like mechanisms [236].

Taken together, these observations underscore the notion that TDP‐43 prion‐like behavior could emerge from a convergence of phase transitions, aggregation, intercellular propagation, and heterotypic interactions with other misfolded proteins, positioning TDP‐43 within a shared pathogenic landscape rather than as an isolated disease driver.

## 6 Therapeutic Strategies Targeting the Prion‐Like Behavior of TDP‐43

**FIGURE 4:**
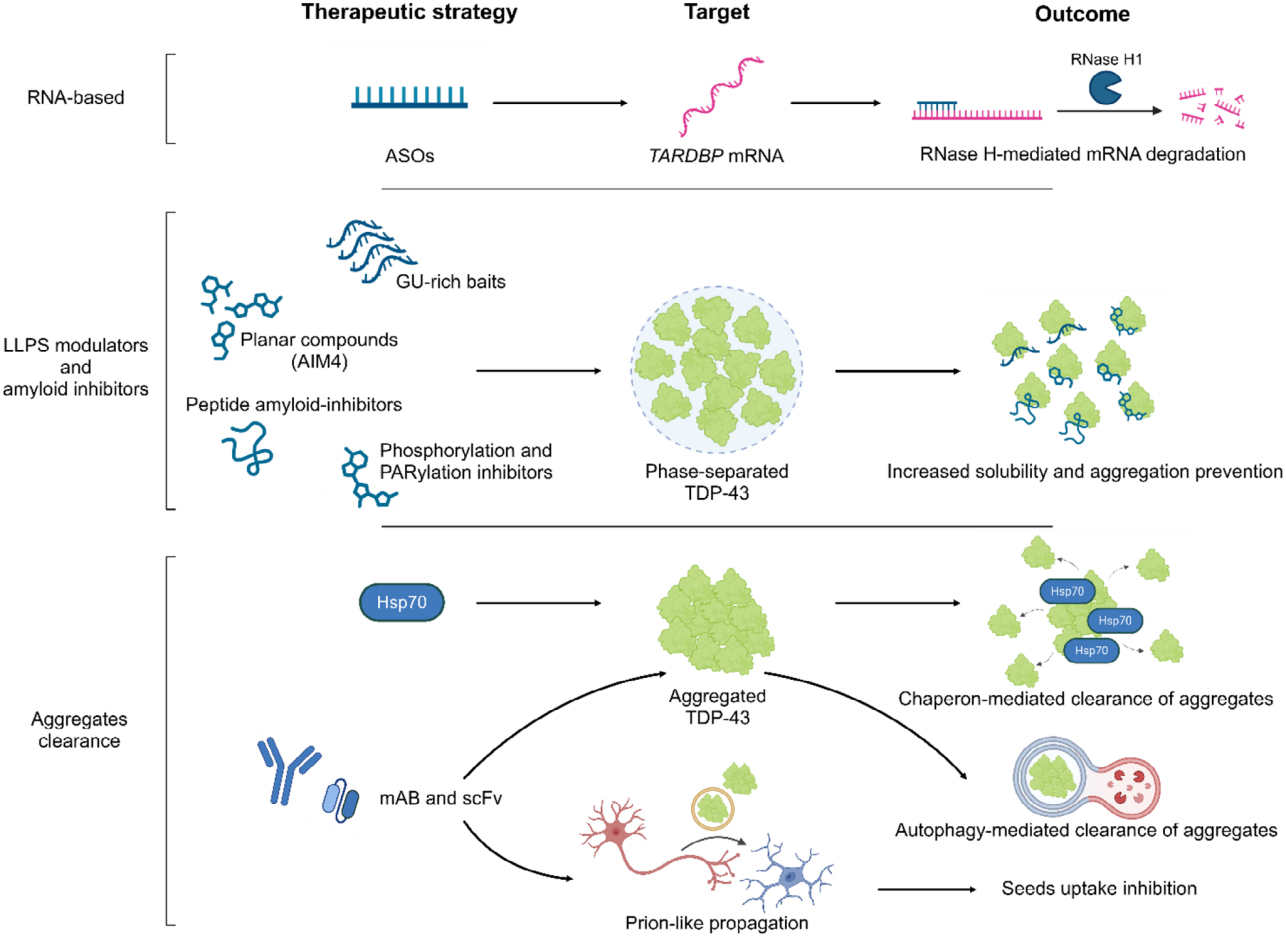
Therapeutic strategies targeting TDP‐43 prion‐like behavior. Schematic overview of current and emerging therapeutic approaches to prevent aberrant TDP‐43 aggregation and propagation. RNA‐based strategies, such as antisense oligonucleotides (ASOs), target *TARDBP* mRNA and promote its degradation via RNase H–dependent mechanisms, reducing TDP‐43 protein levels and preventing excessive phase‐separation. Modulators of liquid–liquid phase separation (LLPS), such as GU‐rich RNA baits, planar compounds, kinases and tankyrases inhibitors, and peptide‐based amyloid inhibitors destabilize aberrant TDP‐43 phase separation and self‐assembly, increasing protein solubility and preventing the formation of pathological aggregates. Aggregate‐directed clearance strategies enhance the removal or neutralization of misfolded TDP‐43 species through chaperone‐mediated strategies (e.g., Hsp70) and immunotherapeutic approaches, including the use of monoclonal antibodies (mAb) and single‐chain Fragment variable (scFv), mediate the removal of misfolded and aggregated TDP‐43 through autophagy activation. Immunotherapy could additionally prevent the cell‐to‐cell spreading of TDP‐43 pathology by inhibiting the uptake of TDP‐43 extracellular seeds by healthy cells. Created in https://BioRender.com.

Currently there are no drugs targeting TDP‐43 directly in ALS or FTLD [111]. The fact that TDP‐43 is ubiquitously expressed and regulates a myriad of RNA‐related events within the cells, makes it a difficult target to build a therapeutic approach. In addition, TDP‐43 toxicity relies not only on the pathological aggregates, but also on its prion‐like behavior which impacts on its spreading and propagation capabilities. For this reason, although an effective treatment is still missing, therapeutic approaches against prion diseases could be repurposed for TDP‐43 proteinopathies. The main strategies against prion diseases involve the downregulation of PrP^C^ through antisense oligonucleotides (ASOs) or RNAi, aggregates clearance, and immunotherapies [237]. The following paragraphs will provide an overview of the possible strategies against TDP‐43 focused on its prion‐like behavior (Figure 4).

### 6.1 ASOs

In the last decade, RNA therapeutics offered great support for neurodegeneration [238]. Two ASOs were indeed approved to treat patients with motor neuron diseases which are nusinersen for spinal muscular atrophy (SMA) [239] and Tofersen for SOD1 ALS [240], while another one (Jacifusen) targeting FUS in ALS patients is currently under clinical trial [241]. ASOs can mediate RNA interference and promote the degradation of the target RNA, thus lowering the levels of the target protein without editing the DNA sequence. Considering that high TDP‐43 levels can initiate its phase separation, ASOs may be useful to decrease TDP‐43 levels, which could slow down the progression of the disease. Being highly negative charged, ASOs cannot cross the blood‐brain barrier, but they can be administered by intrathecal delivery, which avoids immunogenicity issues. This has been supported by an in vivo study conducted in a transgenic mouse model expressing human TDP‐43. A single intracerebroventricular injection of ASOs against the *TARDBP* mRNA reduced TDP‐43 cytoplasmic aggregation and improved the mice behavioral phenotypes, with a dose‐dependent knockdown [242]. However, reduction in TDP‐43 levels must be controlled, as full TDP‐43 depletion is highly detrimental and was demonstrated to be embryonic lethal [243]. With this in mind, in the presence of disease‐associated TDP‐43 mutations, it may be more suitable to use allele‐specific ASOs against the mutant TDP‐43, therefore leaving the wild‐type copy functional (although in this case it will be difficult to find an ASO that only targets specifically the mutated nucleotide sequence compared to the normal one, as with most of TDP‐43 mutations the change will just be represented by a single‐nucleotide substitution).Nonetheless, efforts in this direction have already been tried in the past [244].

### 6.2 Chaperons

Alterations in protein homeostasis are prominent drivers of toxicity in neurodegenerative diseases, and the accumulation of misfolded proteins may be mitigated by enhancing molecular chaperone activity of Heat shock proteins (Hsps), particularly members of the Hsp70 family, represent the most common chaperon proteins, promoting protein folding, preventing protein aggregation and facilitating the clearance of misfolded proteins [245]. Several Hsps are dramatically downregulated in ALS transgenic mouse model and in ALS spinal cord lysates, suggesting impaired proteostasis in the affected tissues [246]. Confirming this, the overexpression of Hsp70 and DnaJ Heat Shock Protein (Hsp40) member 2 in HEK‐293 cells was proven to increase the clearance of TDP‐43 aggregates in a proteasome‐ and autophagy‐independent manner [246].

These observations provide a mechanistic rationale for therapeutic strategies that aim to boost Hsps activity to eliminate pathological aggregates in ALS. However, the recent failure of the phase III clinical trial of arimoclomol, an inducer of Hsp70, suggests that systemic overstimulation of the HSR may not be sufficient to obtain a therapeutical outcome in neurogenerative diseases [247].

### 6.3 Modulation of LLPS and Peptide‐Based Inhibition of Aggregation

Considering the nature of prion aggregates, therapeutic strategies addressing prion diseases do not consider the role of LLPS. However, given the crucial role of phase‐separated RBPs in ALS and FTLD, modulation of LLPS has gained an increased attention.

Since LLPS can be physiologically tuned in several ways, multiple approaches can be employed to target TDP‐43 phase separation. First, as already discussed, GU‐rich bait oligonucleotides antagonize TDP‐43 phase separation, excluding it from the condensate and favoring its solubilization by competitive sequestration by the baits [141]. In parallel, modifications of PTMs constitute a powerful axis for LLPS regulation, especially considering the aberrant PTMs occurring in TDP‐43 aggregates. Therefore, pharmacological targeting of PTM enzymes acting on TDP‐43 could modulate TDP‐43 phase separation and consequently its aggregation. At present, the best target to be considered could be represented by the aberrant phosphorylation of TDP‐43.

TDP‐43 can be phosphorylated by several kinases, including CK1, CK2, CDC7, TTBK1, TTBK2, and GSk3b [48, 248, 249]. The CK1δ inhibitor IGS‐2.7 was shown to induce neuroprotective effects by reducing the phosphorylation of TDP‐43 fragments in both ALS transgenic mouse model and patient‐derived cells, providing promising preclinical data [249, 250]. Moreover, IGS‐2.7‐treated sALS lymphoblasts released a lower amount of EVs in the culture media compared to control cells, suggesting also a role in preventing TDP‐43 pathology propagation [225]. Regarding GSK3b, its activity is increased in sALS lymphoblasts, accompanied by an increase in TDP‐43 phosphorylation [251]. Treatment of these cells with Tideglusib, which is a GSK3b inhibitor, was able to ameliorate the aberrant phosphorylation and reduce the number of cytoplasmic TDP‐43 inclusions [251]. Moreover, inhibition of GSK3b with CHIR99021 was recently proven to reduce the caspase‐dependent formation of CTFs and to improve the survival of human iPSC‐derived forebrain neurons [248]. However, we should keep in mind the observation that hyperphosphorylation of TDP‐43 could inhibit aggregation, raising some concerns about reducing phosphorylated TDP‐43 in human patients [143]. Nonetheless, this evidence points out that regulation of kinases can impact on TDP‐43 pathology on different levels and not only at the phase separation one.

Beyond phosphorylation, PARP‐dependent PARylation has emerged as a potential therapeutic route in TDP‐43 proteinopathies, since it was shown to promote the cytoplasmic translocation of TDP‐43 with subsequent SGs formation [158, 159]. Moreover, motor neurons from ALS patients’ spinal cord exhibit high levels of PAR in the nucleus, suggesting an increased PARP activity [252]. In keeping with expectations, inhibition of nuclear PARP‐1/2 and Tankyrase‐1/2, using Veliparib and XAV939 respectively, was able to reduce the formation of cytoplasmic TDP‐43 aggregates in cultured cells without affecting SGs formation [252].

On a different approach, several molecules have been identified to prevent the recruitment of TDP‐43 within SGs. Particularly, planar compounds, such as mitoxantrone and cycloheximide, were shown to reduce TDP‐43 recruitment inside SGs and to decrease persistent cytoplasmic TDP‐43 inclusions in human iPS‐derived motor neurons [253, 254]. This effect is mediated uniquely by their planar morphology, whose aromatic groups disrupt the stacking π‐π interactions formed within the droplet. Moreover, the downregulation of Ataxin2 (ATXN2), which is a core component of SGs, decreased the levels of CTFs and phosphorylated TDP‐43 aggregates in mice and prevented both SGs maturation and TDP‐43 recruitment in U2OS cells [255]. In this respect, however, the recent failure of a clinical trial aimed to reduced Ataxin levels in patients (ALSpire) did not yield the expected results and was terminated, further highlighting the complexity of targeting a unique disease modifier in such complex disease as ALS.

Acridine derivatives such as AIM4 were shown to significantly prevent TDP‐43 oligomers maturation into amyloid fibrils without altering protein expression levels [256]. Computational analysis suggested that AIM4 is mainly interacting with 288–319 amino acidic region of PrLD, especially residues G288 and F289 which were proven to be crucial for TDP‐43 LLPS [257]. Indeed, AIM4 could antagonize the phase separation of TDP‐43 A315T mutant fragments [257].

Peptide drugs have emerged as potential strategy to block the formation of aggregates by directly targeting the structural interfaces that govern amyloid assemblies. Peptide‐based amyloid inhibitors can be designed according to the following strategies: directly from amyloidogenic cores, engineering cross‐amyloid interactions, exploitation of interactions with chaperones or non‐amyloidogenic peptides, and through peptide libraries screening [258]. Focusing on the first approach, peptides can derive directly from the aggregation‐prone segment of the protein or designed to sterically interfere with the amyloid formation [258]. In both cases, by exploiting sequence and structure specific features of amyloid cores, such peptides can bind the defined interfaces within the amyloids, thus interfering with the assemblies of pathological misfolded proteins. This approach has already been extensively explored in Alzheimer's disease. For instance, a peptide coming from amyloid β 40, termed Aβ(16‐20), fused to oligo‐Glu or Lys tags, strongly inhibited amyloid‐mediated cytotoxicity [259]. At present, the therapeutic relevance of peptide‐based inhibitors in ALS is still hypothetical, nevertheless their characteristic of interfering with the amyloid architecture is highly compatible with TDP‐43 proteinopathy.

While these approaches provide valuable insights into potential therapeutic approaches, it is important to note that many of them are currently at a preliminary stage. Pharmacological modulation of LLPS has been largely validated in in vitro systems, with limited evidence in physiologically relevant models. Moreover, targeting kinases involved in TDP‐43 phosphorylation may lead to context‐dependent and sometimes opposing effects, reflecting the complex and not fully understood role of post‐translational modifications in TDP‐43 biology. Similarly, strategies aimed at modulating stress granules are still far from clinical application due to their essential roles in cellular stress responses. Taking all in consideration, therapeutic interventions targeting LLPS or associated pathways must carefully consider potential toxicity, as they may interfere with fundamental cellular processes. Further studies are essential to better define the specificity, safety, and translational potential of these approaches.

### 6.4 Antibody‐Mediated Aggregate Clearance

Antibody‐based approaches in ALS could offer multiple modes of action, including direct engagement of the aggregates, neutralization of extracellular seeds, and enhancing their microglial clearance. Rather than active immunization, immunotherapies against PrLD should be preferred for TDP‐43 proteinopathies, which have proven great efficacy in preclinical settings. For example, the monoclonal antibody (mAb) ACI‐6677, which binds to 274–320 aa residues within the protein‐resistant amyloid core of TDP‐43, prevented not only TDP‐43 aggregation in vitro, but it also improved the microglial clearance of the aggregates in vivo, and reduced phosphorylated TDP‐43 levels in both injection site and contralateral regions [260]. Antibody biodistribution could be further implemented with single‐chain fragment variable (scFv). For instance, a scFv was generated from the 3B12A mAb which recognizes the residue D247 within NES, which is usually masked in physiological conditions [261]. In HEK293 cells, transfection of this 3B12A scFv increased the proteasome‐mediated clearance of TDP‐43 aggregates [261]. The scFv efficacy was further improved with the addition of a chaperon‐mediated autophagy (CMA)‐signal, which induces HSP70 transcription that contributed to the aggregate clearance [261].

Another mAb targeting the PrLD, termed ACI‐5891, exhibited multiple mechanisms of action: it decreased the levels of phosphorylated and insoluble TDP‐43 both in vitro and in vivo, while extracellularly it prevented the seed uptake from healthy neurons and increased their microglial phagocytosis [262].

In conclusion, thanks to their potential pleiotropic effects, the antibody‐based approaches against TDP‐43 PrLD have the possibility to reverse the progression of the disease instead of just slowing it.

### 6.5 Autophagy Promoters

Degradation of the aggregates could be obtained also by directly inducing autophagy enhancement. Autophagy is a process in which cells degrade their own components through the lysosomal system. Autophagy acts on organelles, misfolded or aggregated proteins, allowing the recycling of the breakdown products [263]. However, dysregulated autophagy can lead to apoptosis, which can contribute to several diseases, including neurodegenerative ones. For example, treatment with rapamycin, which is the inhibitor of the autophagy inducer mTOR, showed a reduction in CTFs accumulation in cultured cells as well as in mice, together with a rescue of motor neuron loss and cognitive defects [264, 265]. The promising results of rapamycin led to the start of the RAP‐ALS clinical trial, which however was concluded at phase II clinical trial due to a lack of therapeutic effects on patients, although it has to be acknowledged that they were in a very reduced number [266]. More recently, additional evidence supporting autophagy enhancement as potential therapeutic approach comes from Abemaciclib and Vacuolin‐1, which decreased TDP‐43 accumulation by promoting omegasome and autolysosome formation in SH‐SY5Y cells [267].

## 7 Conclusions

TDP‐43 has emerged as a paradigm for understanding how the same physicochemical principles that enable dynamic biomolecular organization can also predispose to neurodegeneration. Its ability to occupy multiple polymeric and phase‐separated states underlies a delicate functional balance: reversible self‐assembly is essential for RNA metabolism and the formation of MLOs, yet the same molecular features render TDP‐43 inherently vulnerable to aberrant solidification and aggregation. This duality highlights a central concept in protein homeostasis: physiological and pathological assemblies are not distinct phenomena, but rather interconnected states along a shared continuum.

Importantly, growing evidence suggests that TDP‐43 pathology cannot be explained solely by the presence of aggregates but instead reflects a multifactorial process involving not only impaired clearance, aberrant subcellular localization, and disruption of functional interaction networks, but also altered material properties. In this context, pathological TDP‐43 conversion should be viewed not as a single event, but as the progressive breakdown of mechanisms that normally preserve assembly reversibility and proteostatic control.

A major challenge for the field will be to define which TDP‐43 species are truly neurotoxic, which represent inert end‐stage deposits, and which may instead constitute protective buffering states. Resolving this issue will be critical for therapeutic development, as interventions aimed at broadly suppressing TDP‐43 self‐assembly may inadvertently compromise its physiological functions. Future strategies will therefore need to selectively target pathological conformations or aberrant phase transitions while preserving the dynamic and reversible assemblies required for normal cellular activity.

Ultimately, continued integration of polymer physics, structural biology, cell biology, and neurodegeneration research will be essential to dissect the molecular determinants governing TDP‐43 state transitions. Such interdisciplinary efforts will not only advance understanding of TDP‐43 proteinopathies, but may also provide a broader framework for interpreting the role of aberrant phase transitions in neurodegenerative disease.

## Author Contributions

**Luca Zangrando**: conceptualization, investigation, writing – original draft. **Emanuele Buratti**: conceptualization, writing – review and editing, supervision. **Francesca Paron**: conceptualization, writing – review and editing, supervision.

## Funding

This study was supported by AriSLA 2022‐NOSRESCUEALS.

## Conflicts of Interest

The authors declare no conflicts of interest.

## Data Availability

Data sharing not applicable to this article as no datasets were generated or analysed during the current study.
